# Global investigation of an engineered nitrogen-fixing *Escherichia coli* strain reveals regulatory coupling between host and heterologous nitrogen-fixation genes

**DOI:** 10.1038/s41598-018-29204-0

**Published:** 2018-07-19

**Authors:** Zhimin Yang, Yunlei Han, Yao Ma, Qinghua Chen, Yuhua Zhan, Wei Lu, Li Cai, Mingsheng Hou, Sanfeng Chen, Yongliang Yan, Min Lin

**Affiliations:** 1 Key Laboratory of Plant Pathology of Hubei Province, College of Plant Science and Technology, Huazhong Agricultural University, Wuhan, 430070 China; 2 National Key Facility for Crop Gene Resources and Genetic Improvement, Biotechnology Research Institute, Chinese Academy of Agricultural Sciences, Beijing, 100081 China; 3 State Key Laboratory of Agrobiotechnology and College of Biological Science, China Agricultural University, Beijing, 100193 China

## Abstract

Transfer of nitrogen fixation (*nif*) genes from diazotrophs to amenable heterologous hosts is of increasing interest to genetically engineer nitrogen fixation. However, how the non-diazotrophic host maximizes opportunities to fine-tune the acquired capacity for nitrogen fixation has not been fully explored. In this study, a global investigation of an engineered nitrogen-fixing *Escherichia coli* strain EN-01 harboring a heterologous *nif* island from *Pseudomonas stutzeri* was performed via transcriptomics and proteomics analyses. A total of 1156 genes and 206 discriminative proteins were found to be significantly altered when cells were incubated under nitrogen-fixation conditions. Pathways for regulation, metabolic flux and oxygen protection to nitrogenase were particularly discussed. An NtrC-dependent regulatory coupling between *E*. *coli* nitrogen regulation system and *nif* genes was established. Additionally, pentose phosphate pathway was proposed to serve as the primary route for glucose catabolism and energy supply to nitrogenase. Meanwhile, HPLC analysis indicated that organic acids produced by EN-01 might have negative effects on nitrogenase activity. This study provides a global view of the complex network underlying the acquired *nif* genes in the recombinant *E*. *coli* and also provides clues for the optimization and redesign of robust nitrogen-fixing organisms to improve nitrogenase efficiency by overcoming regulatory or metabolic obstacles.

## Introduction

In nature, a variety of genes and islands can be rapidly and frequently horizontally transferred among bacteria, resulting in the acquisition of certain properties such as nitrogen fixation, antimicrobial resistance and pathogenesis, which help bacteria to succeed in altered habitats or new niches^1–3^. However, newly acquired genes or islands become a burden for bacteria if they are not properly integrated with host regulatory systems. A greater understanding of the physiological alterations that occur in an engineered cell following the insertion of large fragments of foreign DNA, especially from distant species, is needed to pave the way towards the goal of biological engineering.

Biological nitrogen fixation is catalyzed, in most cases, by the molybdenum nitrogenase encoded by a highly conserved *nifHDK* gene cluster. Previous studies have shown that the *nif* genes encoding active nitrogenase can be transferred to non-nitrogen-fixing prokaryotes to impart the ability to reduce atmospheric nitrogen gas into ammonia as a nitrogen source^4–12^. From the perspective of synthetic biology, one key goal of studying biological nitrogen fixation is to facilitate the introduction of this ability into organisms of great importance for human beings, for instance, engineering autonomous nitrogen-fixing cereal crops^13–15^. Eventually, a successfully engineered N_2_-fixing non-legume crop may significantly cut down the use of chemical fertilizers for a cleaner environment and higher yields^16,17^.

In recent years, the synthesis of nitrogen-fixing systems has become increasingly common due to advances in synthetic biology. Normally, a functional entity or pathway is first detected in prokaryotes before being transferred to eukaryotes and plants. Because of its well-studied genetic background, *Escherichia coli* is frequently used as the preferred first-step research model. Following the pioneering work on nitrogen-fixation engineering in 1970s^4,5^, several groups have reported successful gene transfer of *nif* genes to *E*. *coli* in the past five years^11,12,18,19^. However, these recombinant *E*. *coli* stains showed much lower nitrogenase activity compared with the original host^11,12^^,^, and the horizontally acquired ability was insufficient to enable diazotrophic growth on nitrogen-free medium, implying the presence of (i) regulatory coupling between the host and heterologous nitrogen-fixation systems, as well as (ii) a regulatory/or metabolic barrier that results in reduced nitrogenase activity in the engineered cells. To date, how the non-diazotrophic host maximizes opportunities to fine-tune the acquired capacity for nitrogen fixation has not yet been fully explored.

*Pseudomonas stutzeri* A1501 is a root-associated bacterium that exhibits an unusual feature, for a *Pseudomonas* strain, the ability to fix nitrogen^20–24^. The *P*. *stutzeri* A1501 genome contains a 49-kb nitrogen fixation island (NFI) that comprises the largest group of *nif* genes identified to date^25^. Within this island, a total of 52 *nif*-related genes are organized into 11 putative NifA-δ^54^-dependent operons^24^. *nif* gene expression in A1501 was revealed to be tightly regulated at both the transcriptional and post-transcriptional levels^22,23,26,27^. Given its natural integrity and well-studied regulation, the A1501 NFI is a promising model for studying the synthetic biology of nitrogen fixation systems.

We previously transferred the entire *P*. *stutzeri* A1501 NFI into *E*. *coli* and found that the nitrogenase activity of the engineered *E*. *coli* strain was dependent on the external ammonium concentration, oxygen tension and temperature^12^. Similar to previous reports, the nitrogenase activity of recombinant *E*. *coli* strain EN-01 was much lower than that of A1501. In the present study, to better understand the global regulatory effect of the host on NFI expression, we monitored the global transcriptional and proteomic profiles of recombinant *E*. *coli* grown anaerobically under nitrogen-fixation and nitrogen-repression conditions. Furthermore, the metabolic flux shift of *E*. *coli* EN-01 under nitrogen-fixation conditions was also determined by HPLC. To the best of our knowledge, this is the first report of a global investigation into the regulatory cascade of *nif* genes in an engineered nitrogen-fixing organism. These data are particularly useful for providing a more comprehensive understanding of how the *E*. *coli* host intervenes in the transcriptional regulation of the “foreign” NFI to support a functional nitrogenase complex. Our results will also help guide efforts to more successfully remodel and optimize similar systems in other species.

## Results

### Overview of the *E*. *coli* EN-01 transcriptome and proteome under nitrogen-fixation and nitrogen-repression conditions

**Figure 1:**
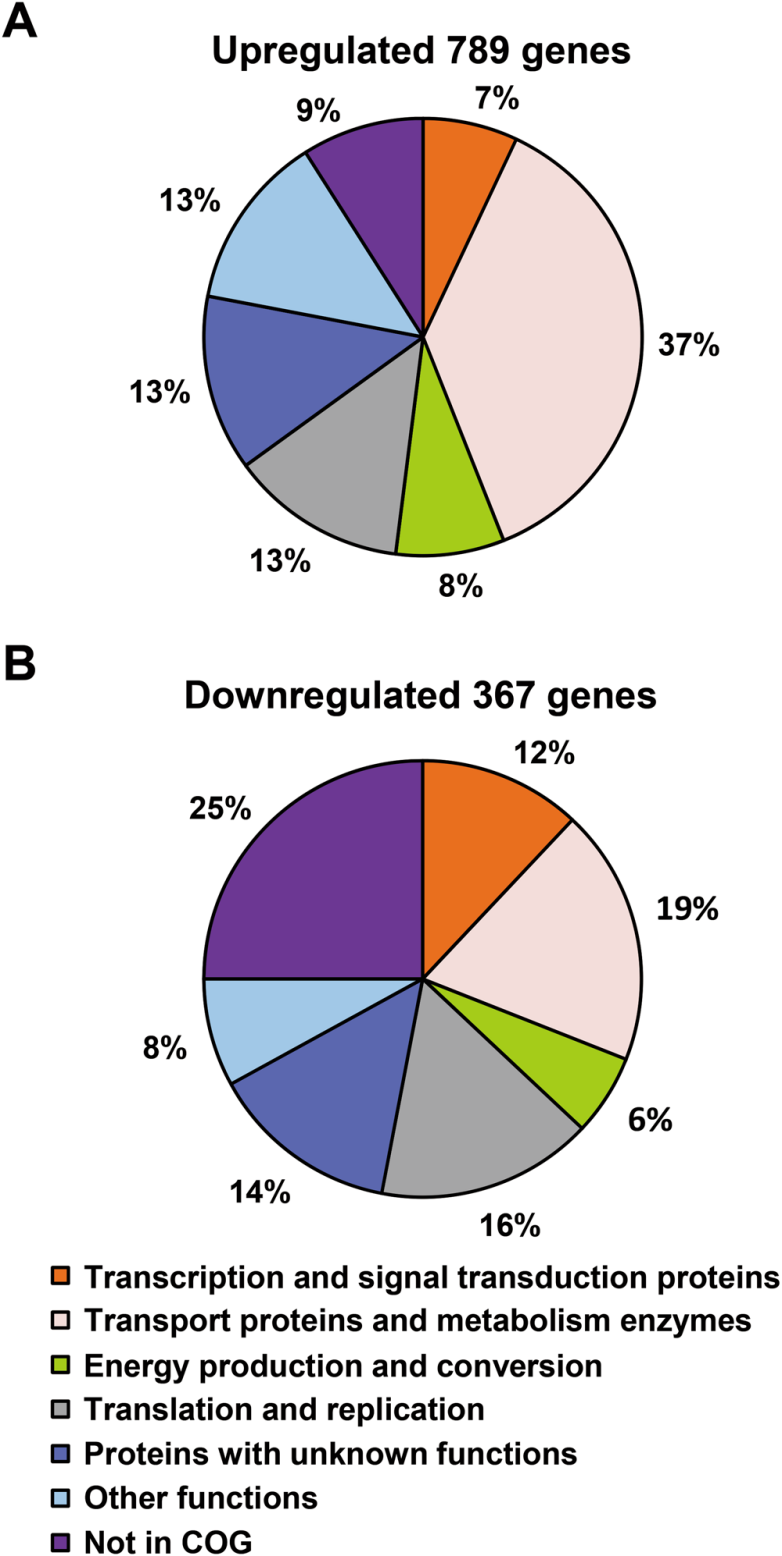
Overview of expression profiling analysis in recombinant nitrogen-fixing *E*. *coli* EN-01. (**A**)Functional categories of nitrogen fixation-induced genes (P ≤ 0.05 and fold change ≥2) in *E*. *coli* EN-01. (**B**) Functional categories of core subset of down-regulated genes (P ≤ 0.05 and fold change ≥2) under nitrogen-fixation conditions. The percentage of genes in each section is depicted.

The expression of 1156 genes was significantly altered (≥2-fold, P ≤ 0.05) under nitrogen-fixation conditions compared with nitrogen-repression conditions, including 789 up-regulated and 367 down-regulated genes. The up-regulated genes mainly belonged to three major functional categories: nitrogen metabolism, transport or membrane protein and unknown function, while the down-regulated genes were mainly involved in energy synthesis, transport, protein synthesis and regulation. These altered genes were further classified according to the COG functional classification system. As shown in Fig. 1, 259 genes, of which 102 were down-regulated and 157 were up-regulated, were involved in bacterial preservation and processing of genetic information (DNA replication, duplication, repair and gene transcription, expression, etc.). A total of 361 genes (31% of the total altered genes) were involved in transport and metabolic pathways. An additional 90 genes were involved in energy synthesis and transformation processes, with 67 genes induced under nitrogen-fixation conditions (Fig. 1A). Moreover, the expression of 151 genes encoding proteins of unknown function was also significant altered.

Transcriptome analysis has revealed that 255 genes were significantly up-regulated and 294 genes were severely down-regulated in *P*. *stutzeri* A1501 under nitrogen-fixation conditions^25^. Subsequent comparison of the *E*. *coli* EN-01 and *P*. *stutzeri* A1501 transcriptomes revealed that at least 24 genes in the *E*. *coli* EN-01 transcriptome showed a similar expression pattern to that in *P*. *stutzeri* A1501 (Supplementary Table S1), including the *glnK*-*amtB* operon, the two-component regulatory system *ntrBC* andthe serine protein kinase-coding gene *prkA*, implying that the overlapping genes or systems might be essential for NFI expression in *E*. *coli*.

Proteomic analysis of *E*. *coli* EN-01 was also performed under identical conditions. A total of 110 protein spots increased by more than 2-fold under nitrogen-fixation conditions; notably, 55 proteins were only expressed under nitrogen-fixation conditions. In addition, 96 proteins were significantly down-regulated under nitrogen-fixation conditions, and the expression of 24 protein spots was abolished on the 2D-PAGE gel (Supplementary Fig. S1). A total of 138 proteins were further identified by MALDI-TOF mass spectrometry, of which 63 were up-regulated and 75 were down-regulated (Supplementary Table S2). In addition, 132 identified proteins showed similar expression patterns to the transcriptional analysis. These proteins included the glutamine synthetase (GS) GlnA, nitrogen regulatory PII protein GlnK, hydroperoxidase II KatE, oligopeptide ABC transporter OppA, glucose-6-phosphate 1-dehydrogenase Zwf and the dihydrolipoamide dehydrogenase LpdA.

Taken together, the above data revealed dramatic changes to the global transcriptional regulatory networks and cellular metabolic pathways in the recombinant nitrogen-fixing *E*. *coli* EN-01 under nitrogen-fixation conditions. Subsequent analyses focused on the nitrogen regulatory system, energy production and conversion systems, and the oxygen protection-related pathway, which have been reported to be crucial for nitrogen fixation^28^.

### Expression patterns of *E*. *coli* nitrogen metabolic pathways and regulatory system under nitrogen-fixation conditions

#### Expression of *P*. *stutzeri* NFI genes in recombinant *E*. *coli* strain EN-01

**Figure 2:**
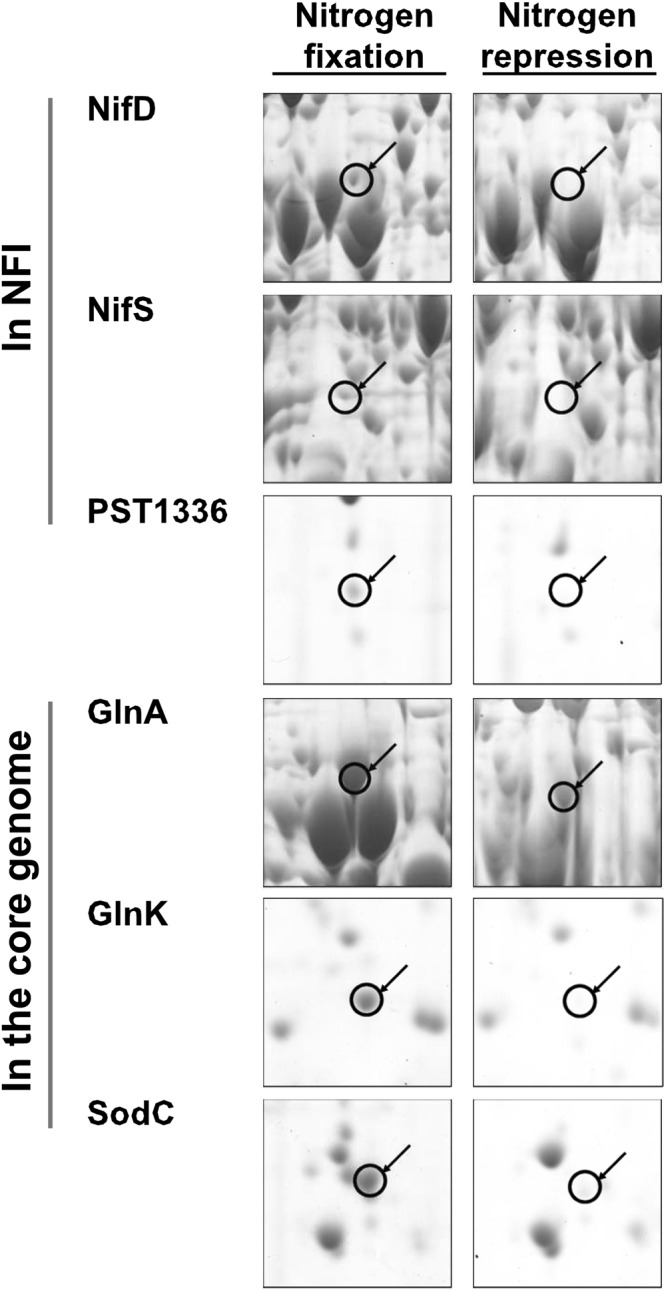
Enhanced expression of nitrogen fixation related proteins in recombinant *E*. *coli* EN-01 under nitrogen-fixation conditions. Magnified regions of 2-D gel images were sliced from Supplementary Fig. S1. Protein spots of interest are indicated with circles with the corresponding protein names pointed out on the left. Proteins were identified by MALDITOF-MS analysis.

EN-01 exhibits nitrogenase activity under glucose-fermentation and nitrogen-fixation conditions, indicating the heterologous production of a functional nitrogenase complex^12^. To further test the heterologous expression of *nif* genes, we determined the induction ratio of 43 genes within the island using quantitative real-time RT-PCR (Table 1). Previously, we found that the A1501 NFI genes are organized into 11 NifA-σ^54^-dependent operons and are up-regulated under nitrogen-fixation conditions^24^. Similarly, except for a 7-gene operon (PST1302-1306), all NifA-σ^54^-dependent operons were strongly induced in *E*. *coli*, suggesting that the *nif*-specific transcriptional activator NifA is controlled by the *E*. *coli* nitrogen regulatory system. Consistent with the RT-PCR results, proteomic analysis also revealed that the NifS, NifD and PST1336 proteins encoded by the NFI genes were up-regulated under nitrogen-fixation conditions (Fig. 2). Why the PST1302-1306 operon (including the *nifQ* and *nifB* genes) was not regulated in EN-01 is still unclear. The *nifQ* mutant of *Klebsiella pneumoniae* is defective in nitrogen fixation due to an elevated requirement for molybdenum^29^. In addition, *nifB* has been long recognized as crucial for nitrogen fixation because NifB participates in the biosynthesis of the FeMo-co factor^30^. We therefore postulate that the relatively low nitrogenase activity of EN-01 might be partly attributed to low expression of these factors within the operon.

**Table 1:** Heterologous NFI gene expression in *E*. *coli* EN-01 under nitrogen-fixation conditions and comparison with the corresponding genes in *P*. *stutzeri* A1501.

Gene ID	Gene name	Up-regulation folds	
		Heterologous expression in *E*. *coli*	Expression in *P*. *stutzeri* A1501^a^
PST1302	PST1302	0.68 ± 0.04	16.83
PST1303	PST1303	0.77 ± 0.12	53.99
PST1304	*nifQ*	1.03 ± 0.14	46.56
PST1305	PST1305	1.11 ± 0.36	38.67
PST1306	*nifB*	0.73 ± 0.22	21.46
PST1313	*nifA*	7.53 ± 0.56	6.95
PST1314	*nifL*	5.58 ± 0.31	7.68
PST1316	*rnfB*	6.8 ± 1.06	9.94
PST1317	*rnfC*	6.59 ± 0.69	2.22
PST1318	*rnfD*	6.57 ± 0.74	7.67
PST1319	*rnfG*	5.96 ± 0.87	7.47
PST1320	*rnfE*	6.02 ± 1	8.80
PST1321	*rnfH*	6.09 ± 0.38	17.55
PST1322	*nifY*	8.67 ± 1.25	21.74
PST1325	PST1325	8.99 ± 0.3	9.68
PST1326	*nifH*	69.51 ± 8.95	94.05
PST1327	*nifD*	32.36 ± 1.79	54.16
PST1328	nifK	43.88 ± 2.37	38.22
PST1329	*nifT*	8.21 ± 0.84	7.82
PST1330	*nifY*	6.24 ± 0.61	8.51
PST1331	PST1331	5.76 ± 0.7	12.55
PST1332	PST1332	5.16 ± 0.47	3.27
PST1333	*nifE*	68.88 ± 6.88	35.82
PST1334	*nifN*	70.35 ± 5.08	13.32
PST1335	*nifX*	70.01 ± 6	37.97
PST1336	PST1336	39.56 ± 5.22	5.06
PST1341	PST1341	14.21 ± 0.82	1.28
PST1342	PST1342	23.42 ± 2.81	3.69
PST1344	PST1344	11.44 ± 0.94	7.16
PST1345	*modC*	11.59 ± 1.23	1.59
PST1346	*modB*	9.08 ± 2.51	2.05
PST1347	*modA*	7.92 ± 0.68	4.12
PST1348	PST1348	6.86 ± 1.02	3.88
PST1350	*nifU*	103.22 ± 10.52	10.77
PST1351	*nifS*	51.33 ± 8.98	16.21
PST1352	*nifV*	41.38 ± 4.1	24.55
PST1353	*cysE*	17.32 ± 1.85	32.98
PST1354	PST1354	11.3 ± 0.6	11.11
PST1355	*nifW*	6.8 ± 0.49	26.04
PST1356	*nifZ*	12.06 ± 1.46	18.17
PST1357	*nifM*	7.42 ± 1.01	18.78
PST1358	*clpX2*	12.67 ± 1.56	8.42
PST1359	*nifF*	16.81 ± 0.69	14.91

^a^Transcriptional ratios of *P*. *stutzeri* A1501 NFI genes were obtained from DNA microarray experiments^25^.

#### Regulatory coupling between the *E*. *coli* general nitrogen regulatory system and the heterologous *P*. *stutzeri nif* island

Regulation of the nitrogen-fixation process in free-living diazotrophs facilitates the stringent control that is necessary to maximize the physiological benefits from diazotrophy^28^. In *P*. *stutzeri* A1501, the nitrogen regulatory cascade comprises the AmtB–GlnK–NtrBC global nitrogen regulation proteins, which sense the nitrogen signal and subsequently control the expression of the *nif*-specific regulatory proteins NifLA^31^. However, the nitrogen regulatory network in *E*. *coli* appears to be an expanded version compared to that in *Pseudomonas*, with one additional PII protein and cascade regulation achieved by a Nac regulatory protein^32,33^. Acquisition of a *nif*-specific regulatory system in the recombinant *E*. *coli* raises the question of nitrogen-fixation efficiency by these two regulatory systems of different evolutionary origins.

**Figure 3:**
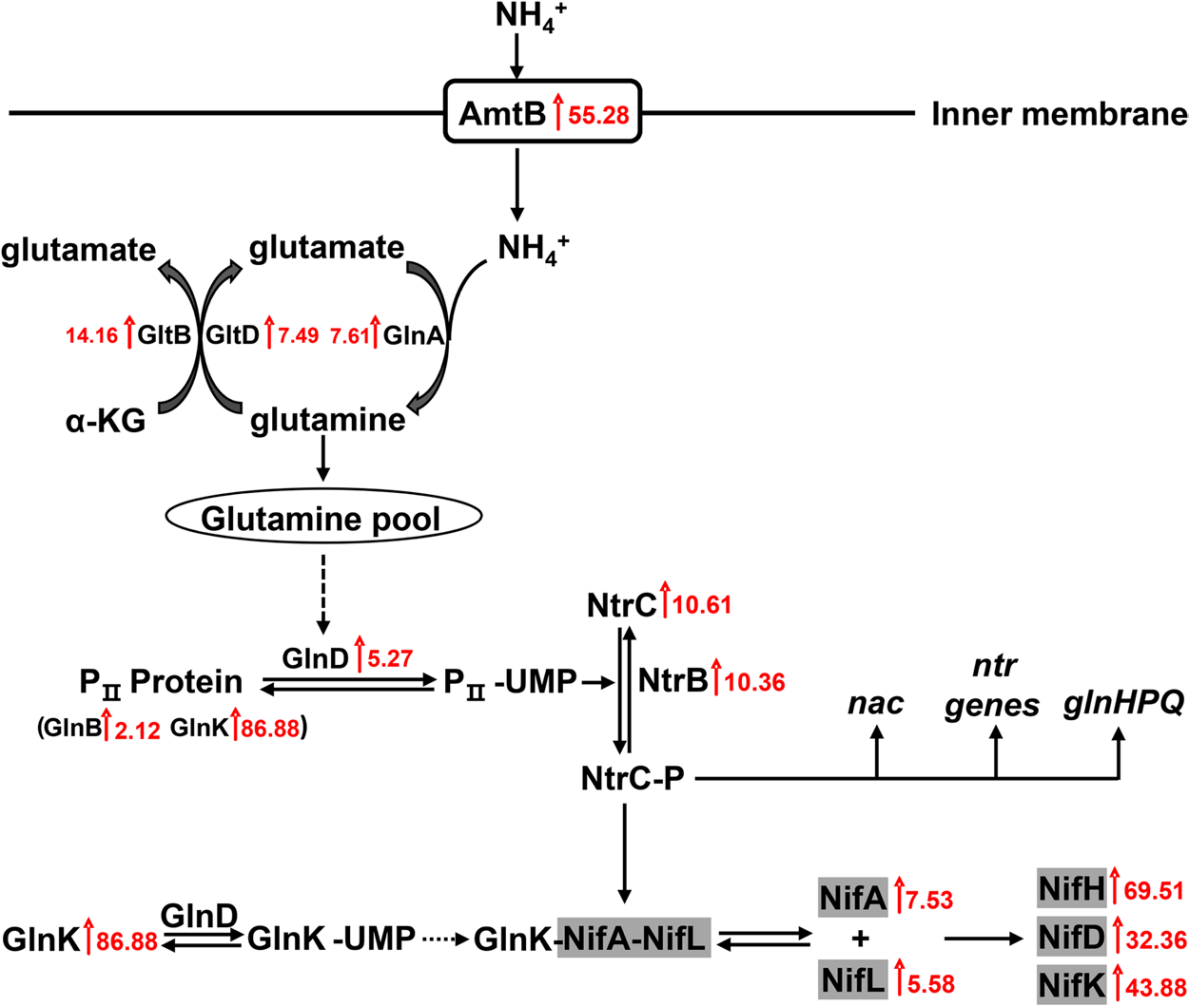
Proposed cascade regulation of *nif* genes in *E*. *coli* EN-01 under nitrogen-fixation conditions. A transcriptional regulatory network was constructed from mRNA and protein expression data. Red arrows, induction. Numbers in red indicates the transcriptional up-regulation ratio of protein- or enzyme-coding genes. Genes with a gray background represent NFI genes. Dashed lines represent predicted regulatory interactions.

Changes in the expression of *E*. *coli* nitrogen regulatory system genes are summarized in Supplementary Table S3. Of these, the *glnLG* genes, which are homologs of *Pseudomonas ntrBC*, were up-regulated by more than 10-fold, the sigma factor *rpoN* was up-regulated by 3.64-fold, the ammonium transporter *amtB* was up-regulated by 55.28-fold, and the two PII protein genes *glnK* and *glnB* were both up-regulated under nitrogen-fixation conditions. Consistent with the transcriptional change, proteomic data indicated that GlnK and GlnA expression was enhanced (Supplementary Table S2). Based on the observations in the present study and those from previous experiments from a wide range of model systems, we propose a regulatory cascade that controls heterologous *nif* transcription in *E*. *coli* EN-01 (Fig. 3): The UTase/URase enzyme GlnD (*glnD* gene was up-regulated 5.27-fold) is activated by low internal glutamine concentrations and further modifies the PII protein GlnB (*glnB* was up-regulated 2.12-fold) and GlnK (*glnK* was up-regulated 86.88-fold) to PII-UMP. Subsequently, PII-UMP phosphorylates NtrC. As a global transcriptional regulator, NtrC activates the expression of a series of nitrogen metabolic genes, including the GS-encoding gene *glnA* (up-regulated 7.61-fold), the nitrogen transcriptional regulation (Ntr) genes, the ammonium transporter gene *amtB* (up-regulated 55.28-fold), the transcriptional regulator *nac* (up-regulated 124.98-fold) and the *nif*-specific regulator genes *nifLA*. In fact, we previously demonstrated cross-complementation of the *P*. *stutzeri* /or *E*. *coli ntrC (glnG)* mutant by the *E*. *coli* /or the *P*. *stutzeri ntrC* gene^34^. We have also shown that the purified *E*. *coli* NtrC protein can bind specifically to the *nifLA* operon promoter region^12^. In a sense, this working model supports a similar expression pattern for the NFI genes in *E*. *coli* with that observed in *P*. *stutzeri* A1501, in that the *nif* genes were subject to regulation by the NtrC protein via the *nif*-specific regulator NifA, suggesting a regulatory coupling of these two different evolutionary systems through a direct activating interaction.

**Figure 4:**
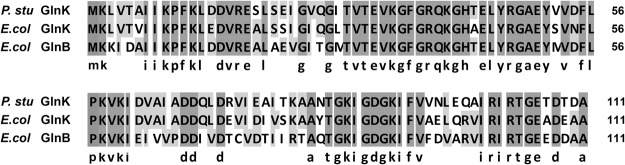
Clustal Omega alignments of PII proteins from *P*. *stutzeri* and *E*. *coli*. Alignments were constructed using DNA Man and refined manually. Consensus sequences with ≥60% identity are reported below the alignment.

The PII protein is recognized as a critical signal transduction protein in bacterial nitrogen metabolism^35^. *E*. *coli* encodes two PII counterparts, GlnB and GlnK, which show 68% and 78% identity, respectively, with the sole A1501 PII protein GlnK and share 67% identity with each other (Fig. 4). Under nitrogen-fixation conditions, *E*. *coli* EN-01 *glnK* mRNA expression was increased by 86.88-fold, while the *glnB* gene was up-regulated by 2.12-fold. GlnK protein was only detected under nitrogen-fixation conditions (Fig. 2). In A1501, an interaction between GlnK and the C-terminal domain of NifL was observed using the yeast two-hybrid system^23^, suggesting GlnK-dependent control of NifA activity by NifL. Based on the overall transcription of the nitrogen regulation system in *E*. *coli* EN-01, we conjectured that, as reported before^35,36^, GlnB and GlnK may constitute a two-tiered regulatory system and that their functions may depend on the timing of expression and the levels of accumulation, while *glnK* may play key roles in nitrogen regulation in *E*. *coli* to adjust to perturbations that occur under nitrogen-fixation conditions.

#### Rearrangement of the pathways for nitrogen assimilation and alternative nitrogen sources

Glutamate and glutamine provide approximately 75% and 25% of cellular nitrogen, respectively. Nitrogen assimilation must therefore result in the synthesis of these two nitrogen donors^37^. The GDH pathway, encoded by the glutamate dehydrogenase gene *gdhA*, is generally associated with a nitrogen-rich, aerobic environment, whereas the GS-GOGAT pathway, encoded by the GS gene *glnA* and the glutamate synthase (GOGAT) genes *gltBD*, is often associated with a nitrogen-limited, microaerobic environment^37,38^. Transcriptomic analyses showed that both operons of the GS-GOGAT system were up-regulated more than 7-fold under nitrogen-fixation conditions, however, the *gdhA* gene was down-regulated by 2.83-fold (Supplementary Table S3). In accordance with the mRNA results, we also observed that GlnA was up-regulated 7.61-fold and GdhA, encoding GDH, was down-regulated 9.1-fold in the proteomic analysis (Supplementary Table S2). Clearly, the recombinant *E*. *coli* EN-01 chooses the high-affinity GS-GOGAT pathway rather than the GDH pathway when environmental nitrogen is depleted.

Zimmer *et al*. previously concluded that *E*. *coli* use a range of pathways to scavenge for nitrogen-containing compounds as a first line of defense against nitrogen starvation^39,40^. In this work, we observed a rearrangement of transport capacity by EN-01 under nitrogen-fixation conditions, with alterations of 299 transport or membrane protein genes. Notably, the ammonium transporter gene *amtB*, which forms a transcriptional operon with the PII protein-coding gene *glnK*, showed a 55-fold enhancement (Fig. 3). Glutamate uptake systems, including the *gltIJKL* operon, which encodes a glutamate/aspartate uptake system; the *glnHPQ* operon, which is responsible for a high-affinity glutamine transport system; the *gadC* gene, which encodes a glutamic acid/γ-aminobutyrate antiporter; and the *gltP* gene, which encodes a proton/glutamate-aspartate symporter, were up-regulated under nitrogen-fixation conditions. Alternative nitrogen-transport systems, for instance, lysine, arginine, ornithine, histidine, serine, polyamine, peptide, dipeptide and oligopeptide transporter genes, were all significantly induced. Additionally, the *astABCDE* operon, which is responsible for the degradation of arginine into N_2_-succinyl-L-ornithine, CO_2_ and ammonia, was also up-regulated (Supplementary Table S3).

Nac is a *glnK*-controlled Ntr protein widely existed in enterobacteria^41^. Nac is well known to be involved in regulating the expression of multiple pathways to modulate the levels of important cellular metabolic intermediates and thus integrates nitrogen assimilation with other aspects of metabolism^41–44^. As expected, *nac* gene expression was drastically up-regulated (over 100-fold) under nitrogen-fixation conditions. *gabDTP* (γ-aminobutyrate transport and degradation), *dppABD* (dipeptide transport), and *fklB* (peptidyl-prolyl cis-trans isomerase) gene expression was also increased (Supplementary Table S3). However, *codBA* (cytosine metabolism), *nupC* (nucleoside transport) and *ydcSTUVW* (putative putrescine transport and degradation) gene expression did not appear to be significantly altered. These genes were recognized to be controlled by the *nac* gene in *E*. *coli*^39^. The unclear inconsistent gene expression obtained in this study may provide important clues into the regulatory disturbances of the NFI island by the *nac*-conferred nitrogen stress response in recombinant *E*. *coli* EN-01.

### Metabolic flux shift of *E*. *coli* EN-01 under nitrogen-fixation conditions

**Figure 5:**
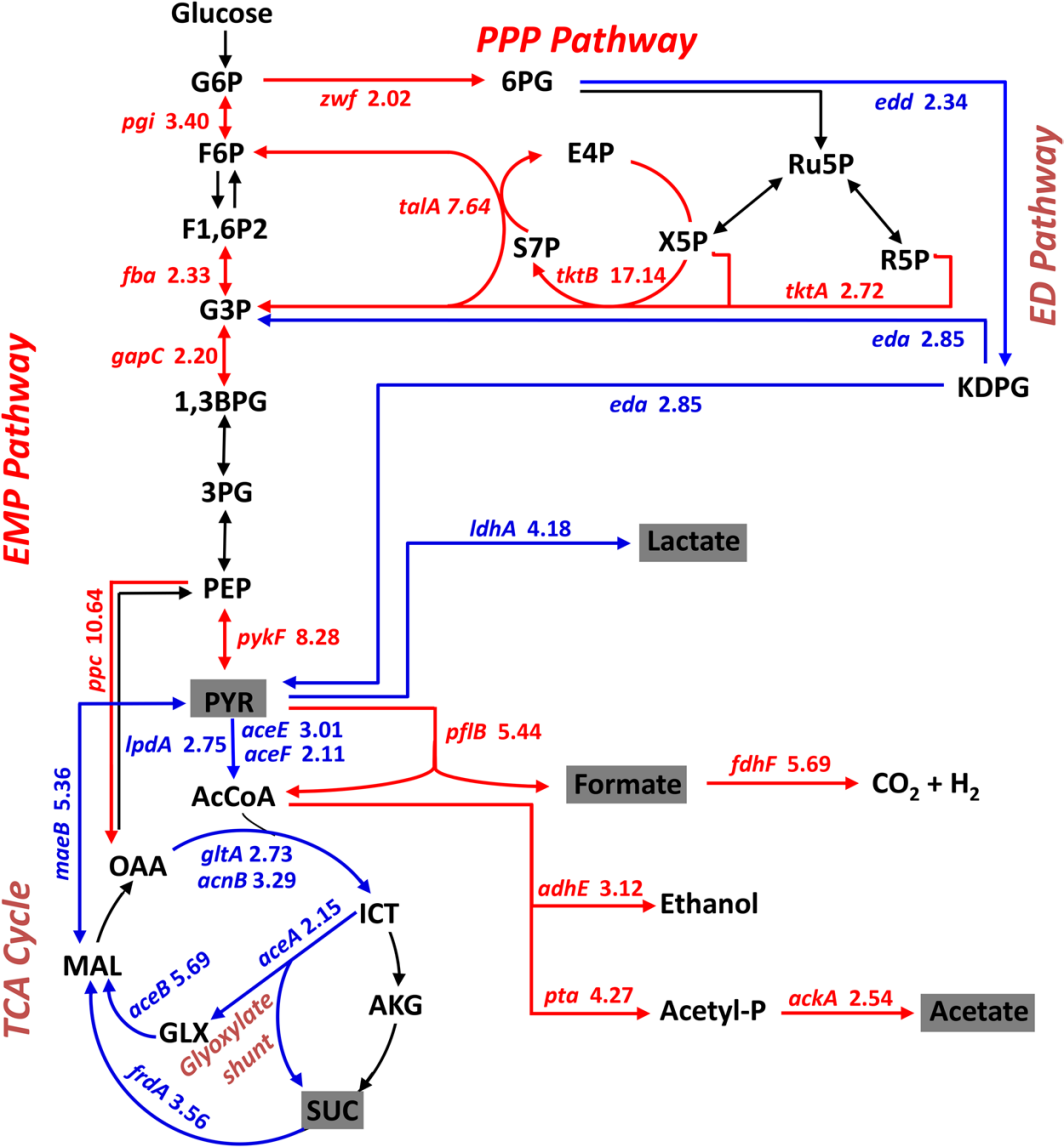
Bioreaction metabolic flux shifts of EN-01 central carbon metabolism under nitrogen-fixation conditions. Changes in the central carbon metabolism network were constructed from microarray data. Arrows indicate the physiological directions of reactions. Red arrows, enhanced expression (P ≤ 0.05 and fold change ≥2); blue arrows, reduced expression (P ≤ 0.05 and fold-change ≥2). The accumulation of organic acids highlighted in gray was detected in the medium by HPLC. Abbreviations: G6P, glucose 6-phosphate; F6P, fructose 6-phosphate; F1,6P2, fructose 1,6-phosphate; G3P, glyceraldehyde 3-phosphate; 1,3-BPG, 1,3 diphosphoglycerate; 3PG, 3-phosphoglycerate; PEP, phosphoenolpyruvic acid; PYR, pyruvate; AcCoA, acetyl coenzyme A; OAA, oxaloacetate; ICT, isocitrate; AKG, α-ketoglutarate; SUC, succinate; MAL, malate; GLX, glyoxylic acid; 6PG, 6-phosphogluconate; E4P, erythrose 4-phosphate; Ru5P, ribulose-5-phosphate; R5P, ribose-5-phosphate; X5P, xylulose -5-phosphate; S7P, sedoheptulose-7-phosphate; KDPG, 2-keto-3-deoxy-6-phosphogluconic acid; Acetyl-P, acetyl phosphate.

Biological nitrogen fixation is an energy-dependent process that requires ATP, produced by the catalytic reaction of carbon sources, to break the N≡N bond^45^. Heterologous NFI expression may place a considerable metabolic burden on the host *E*. *coli*. In the present work, EN-01 was observed to shift its metabolic flux to overcome the energy barrier for nitrogen fixation. As the central carbon pathways constitute the backbone of cell metabolism by providing energy, metabolic building blocks, and reducing power for biomass synthesis, we investigated the responses of central carbon metabolism in EN-01. As shown in Fig. 5, a stronger flux toward the pentose phosphate pathway (PPP) was found under nitrogen-fixation conditions. Thus, the PPP was postulated to serve as the primary route for glucose catabolism, since the key genes encoding the rate-limiting enzymes glucose-6-phosphate 1-dehydrogenase (*zwf*), transketolase (*tktAB*), transaldolase (*talA*) and glucose-6-phosphate isomerase (*pgi*) were highly induced. The enhanced expression of glucose-6-phosphate 1-dehydrogenase was also detected in proteome analyses (Supplementary Table S2). Conversely, the glyoxylate shunt and Entner-Doudoroff pathway were found to be inhibited in EN-01 under nitrogen-fixation conditions. Furthermore, the electron transfer systems commonly involved in energy production^46,47^ were up-regulated, such as the *appABC* (encoding cytochrome *bd*-II oxidase), *hyaABCDE* (encoding the hydrogenase), *fhlA* (encoding the transcriptional activator of formate hydrogenlyase), *fdhF* and *hycABCDEFGH* (encoding formate hydrogenlyase complex), *ndh* (encoding the NADH dehydrogenase), *napH* (encoding the ferredoxin-type protein), and *adhE* (encoding the bifunctional acetaldehyde-CoA/ alcohol dehydrogenase) genes. Meanwhile, both the transcription and translation of the formate acetyltransferase 1 gene *pflB* were enhanced (Supplementary Table S2). Additionally, three alcohol dehydrogenase genes that are reportedly involved in ethanol fermentation were up-regulated.

*E*. *coli* is an anaerobic fermentative bacterium that produces a variety of organic acids by utilizing glucose under nitrogen-fixation conditions; however, these acids are harmful to nitrogenase activity. In contrast to *E*. *coli*, *P*. *stutzeri* A1501 is an alkali-producing strain that supplies an optimal environment for nitrogen fixation at pH 7.0–9.0 (Supplementary Fig. S2). The organic acid content in *E*. *coli* wild-type and EN-01, as well as *P*. *stutzeri* A1501, were determined by HPLC after growth under nitrogen-fixation conditions. *E*. *coli* wild-type and EN-01 produced a large number of organic acids under nitrogen-fixation conditions (Supplementary Table S4), including pyruvic acid, succinic acid, lactic acid, formic acid and acetic acid, whereas A1501 does not generate these organic acids. The presence of these organic acids could certainly have negative effects on nitrogenase activity in the recombinant EN-01 strain. Altering the intracellular acid environment may be a feasible method to increase the nitrogen-fixing ability of *E*. *coli* EN-01.

### Oxygen protection strategy of *E*. *coli* EN-01 to cope with O_2_ toxicity to nitrogenase

Nitrogenase is extremely oxygen sensitive. In fact, purified nitrogenase, regardless of its source, undergoes extremely rapid and irreversible inactivation by O_2_^48^. *E*. *coli* EN-01 can grow under both aerobic and anaerobic conditions but only executes nitrogen-fixing activity anaerobically^12^. Given the importance of oxygen in nitrogen fixation, we explored the potential mechanism for oxygen protection in *E*. *coli* EN-01 under anaerobic nitrogen-fixing conditions.

In the present study, the scavenging capacity of *E*. *coli* EN-01 for superoxide radicals, hydrogen peroxide, and hydroxyl radicals appeared to increase under nitrogen-fixation conditions. The genes *sodC* and *katE*, which encode superoxide dismutase and the hydroperoxidase HPII, were up-regulated 2.94-fold and 15.47-fold, respectively, under nitrogen-fixation conditions (Supplementary Table S3). Furthermore, proteomic analysis revealed these two gene products were increased as well (Supplementary Table S2). Other genes such as the hydrogenase 1-coding genes *hyaABCDEF*, cytochrome *bd*-II synthetic-related genes, which have higher oxygen affinity than ATP terminal oxidase, and all cytochrome *c* synthetic genes, were also up-regulated. The *osmC* gene, whose product is involved in the detoxification of organic hydroperoxides^49,50^, was also up-regulated 5.81-fold under nitrogen-fixation conditions (Supplementary Table S3). In addition, the *yhbO* gene was up-regulated 7.5-fold, and its product was only detected under nitrogen-fixation conditions (Supplementary Table S2). YhbO has been shown to function in the response to various stresses. Mutating the yhbO gene in *E*. *coli* leads to increased sensitivity to heat, oxidative, hyperosmotic, pH and UV stresses^51^.

Although no significant changes were detected at the mRNA level, proteomic analysis revealed that Dps was induced 4.06-fold under nitrogen-fixation conditions (Supplementary Table S2). The Dps protein, a nonspecific DNA-binding protein, is reported to reduce the production of oxidative radicals through ferroxidase activity and therefore to protect the cell from oxidative stress, UV irradiation, iron and copper toxicity, among others^52,53^.

Taken together, the genes or proteins mentioned above, despite their unclear functions, are thought to be involved in the oxygen stress response under nitrogen-fixation conditions and to simultaneously protect the nitrogenase complex from O_2_ inhibition by providing a tolerable oxygen environment in *E*. *coli* EN-01.

## Discussion

To date, the ability to fix nitrogen is exclusively found among bacteria, including green sulfur bacteria, firmibacteria, actinomycetes, cyanobacteria and all subdivisions of proteobacteria^54,55^. Genomic analyses have revealed that nitrogen-fixation genes are clustered in most diazotrophs^56^. The *nif* genes were further presumed to have been lost in most bacterial and archaeal lineages during evolution and that horizontal gene transfer played a pivotal role in the recent acquisition of *nif* genes in some species^55–57^. In agreement with this hypothesis, several groups have found that transferring *nif* genes to non-nitrogen fixers successfully yields functional recombinant nitrogen-fixing strains, albeit with far lower nitrogenase activity compared with the donor strains^5,11,12,18^.

The structural and regulatory systems that control nitrogen fixation in different diazotrophs are extremely well conserved, whereas the regulatory mechanisms of *nif* genes differ from one organism to another^28,54^. Therefore, for recombinant nitrogen-fixing strains harboring a heterologous gene system, a variety of metabolic penalties and regulatory barriers may exist between the two systems. Understanding how to overcome and escape the metabolic penalties and regulatory barriers is essential to fine-tune acquired nitrogen-fixing capacity of the non-diazotrophic host.

In this work, transcriptomic and proteomic analysis separately identified 1156 genes and 138 proteins whose expression was significantly altered when *E*. *coli* EN-01 was incubated under nitrogen-fixation conditions. Our results highlight the metabolic penalties and regulatory barriers for nitrogen signal transduction and metabolism brought by excess fixed nitrogen in the medium. These genes and proteins, particularly the 24 genes that overlapped between the two transcriptomic analyses (the previous transcriptomic analysis of *P*. *stutzeri* A1501 and the *E*. *coli* analysis presented in this work), are potential targets for subsequent modification in *E*. *coli* EN-01 to optimize nitrogen-fixing capacity.

The nitrogen regulatory cascade of *P*. *stutzeri* A1501 comprises the AmtB–GlnK–NtrBC general nitrogen regulation proteins and the *nif*-specific regulatory protein NifLA^22,24,26,31^. *E*. *coli* has a similar nitrogen regulatory system to *P*. *stutzeri* but harbors an additional PII protein and a cascade regulation by the Nac regulatory protein^32,33^. In the present work, a similar expression network of NFI genes in *E*. *coli* to that operating in *P*. *stutzeri* A1501 was predicted based on the expression patterns of *rpoN*, *glnD*, *glnA*, *glnK*, *ntrBC*, *nifLA* and other nitrogen regulation-related genes (Fig. 3). Given that the purified *E*. *coli* NtrC protein can bind specifically to the A1501 *nifLA* operon promoter region^12^, we postulated the regulatory coupling of these two evolutionary divergent systems through a direct activating interaction. Additionally, the *nac* gene was also predicted to be involved in *nif* gene regulation in EN-01 although the mechanism requires further investigation. Interestingly, an expression difference was observed in the two PII counterparts GlnB and GlnK in *E*. *coli*. In principle, the functions of GlnK and GlnB overlap in Enterobacteriaceae. However, both proteins exhibited distinct physiological roles in the regulation of nitrogen assimilation over a wide range of environmental conditions, despite having distinct expression patterns. The *glnB* promoter contains a putative -10 sequence for RpoS activation, while the transcription of *glnK* is induced by NtrC and RpoN^35^. We therefore presumed that *glnK* may play a more important role than GlnB in nitrogen regulation in *E*. *coli* under nitrogen-fixation conditions. Notably, the PST1302-1306 cluster within the NFI showed unregulated expression in *E*. *coli* EN-01 but was highly up-regulated in *P*. *stutzeri* A1501 under nitrogen-fixation conditions^24^. The weak transcription of these nitrogenase component genes may predict imperfections and the necessity for further rebuilding of *nif* gene expression regulation in *E*. *coli* EN-01.

A core challenge for diazotrophs is the need to generate sufficient energy to drive nitrogen fixation. In the present work a strong shift in the metabolic flux of the EN-01 strain was observed under nitrogen-fixation conditions. The key enzyme of the PPP was up-regulated, but the glyoxylate shunt and Entner-Doudoroff pathway were inhibited. Thus, the PPP pathway was concluded to serve as the primary route for glucose catabolism to support nitrogen fixation. Moreover, anaerobic respiration-related electron transfer systems and substances were induced. These data highlight the energy acquisition pathway of EN-01 to adapt to nitrogen fixation. However, as an anaerobic fermentative bacterium, EN-01 produces a large number of organic acids in the media, which may negatively affect nitrogenase activity, since the donor strain *P*. *stutzeri* A1501 is an alkali strain. Changing the intracellular acid environment may thus be a feasible method to increase the nitrogen-fixing ability of *E*. *coli* EN-01.

We identified a set of genes that might be involved in protecting nitrogenase from oxygen via scavenging of superoxide radicals, hydrogen peroxide, and hydroxyl radicals. Two proteins, YhbO and Dps, also appeared to be involved in the oxygen protection of nitrogenase by protecting the cell from oxidative stress. These genes or proteins may protect the nitrogenase complex from O_2_ inhibition by establishing a tolerable oxygen environment in *E*. *coli* EN-01. Therefore, we speculate that improving the expression of antioxidant capacity-related pathway genes may satisfy the need for oxygen protection under microaerobic conditions and perhaps drive the metabolic flux shift from anaerobic respiration to aerobic respiration to produce more energy and reduce the energy requirements of the nitrogen fixation.

The nitrogenase activity of *E*. *coli* EN-01 was much lower than that in *P*. *stutzeri* A1501. According to the data in this work, this difference may be due to the regulatory divergence of nitrogen assimilation, the inactivation of the gene operon PST1302-1306, and the production of organic acids. Another factor may also be involved. In our recent work, a species-specific regulatory ncRNA *nfiS* identified in *P*. *stutzeri* A1501, could directly base paired with the mRNA of the nitrogenase component *nifK* to enhance translation efficiency and transcript half-life, thereby regulating nitrogenase biosynthesis^27^. Blast results showed that no sequence homologous to A1501 *nfiS* was present in the *E*. *coli* genome. Introduction of *nfiS* regulation into *E*. *coli* EN-01 may be a useful way to refine NFI gene regulation.

From an agri-biotechnology perspective, the successful engineering of an N_2_-fixing organism would significantly reduce the need for nitrogen fertilizers^15,16,54^. Biosynthetic techniques have been used by several groups to improve or optimize nitrogenase activity in metabolically engineered strains. Wang X. *et al*. replaced the *nif* regulatory elements in the recombinant *E*. *coli* strain with a T_7_ RNA polymerase–LacI expression system to overcome regulatory barriers, and the newly constructed T_7_-dependent *nif* system bypassed the original complex regulatory circuits with minor physiological limitations^9^. Subsequent work from the same group pinpointed the electron-transfer components from plant organelles that can be used to support nitrogenase activity and reduction of the number of target genes required to engineer nitrogen fixation in plants^58^. Another group observed that five separate gene clusters, as well as a combined cluster from *Paenibacillus* sp. WLY78 and *Klebsiella oxytoca*, increase the nitrogenase activity of the recombinant *E*. *coli* 78-7, which harbors a minimal *nif* gene cluster from *Paenibacillus* sp. WLY78^11,19^.

In summary, the results presented in this work demonstrate the overall expression pattern of *E*. *coli* EN-01 genes under nitrogen fixation and revealed the regulatory coupling of host genes and the heterologous NFI that supports functional nitrogenase activity in the engineered *E*. *coli* strain. We also uncovered the metabolic penalties and regulatory barriers that lead to the reduced nitrogenase activity in *E*. *coli* EN-01 and provided clues for biotechnological applications to generate new hypotheses concerning gene regulation and flux, which may lead to an improved ability to fix nitrogen in *E*. *coli*. This work may shed light on the metabolic penalties and coordinated patterns between host and horizontally transferred genes and provide the knowledge necessary to pave the way for engineered nitrogen fixation.

## Methods

### Bacterial strains and growth conditions

The strains and plasmids used in this study are described in Table S5. The recombinant strain *E*. *coli* EN-01 bearing an NFI was grown at 37 °C in LB medium^12^. Chloramphenicol, tetracycline and hygromycin were added to media at a concentration of 20 μg/mL, 10 μg/mL and 100 μg/mL, respectively, as required. *P*. *stutzeri* A1501 was cultured at 30 °C in LB medium or minimal lactate-containing medium (medium K) as described previously^20^.

### Nitrogenase activity assays

To measure nitrogenase activity in *E*. *coli* EN-01, cells were cultured overnight to an OD_600_ of 2.0 at 37 °C in LB, and 0.2 mL cultures were directly added to 6 mL NFDM minimal medium (20 g glucose, 0.7 g MgSO_4_·7H_2_O, 25 mg Na_2_MoO_4_·H_2_O, 3.6 g ferric citrate, 68 g KH_2_PO_4_, 241 g K_2_HPO_4_, L^−1^, pH 7.4) without nitrogen in 10 mL serum bottles. These cultures were sealed and then incubated at 30 °C for 20 h with constant shaking (200 rpm), after which 10% acetylene without oxygen was injected to determine ethylene production by gas chromatography, as described by Cannon *et al*.^59^. The nitrogenase activity of *P*. *stutzeri* was determined in N-free minimal medium at an OD_600_ of 0.1 at 30 °C under an argon atmosphere containing 0.5% oxygen and 10% acetylene, according to the protocol described by Desnoues *et al*.^60^. Nitrogenase-specific activity is expressed as nmol ethylene per mg protein per h. Each experiment was repeated at least three times.

### Total RNA preparation

*E*. *coli* EN-01 cells were cultured overnight to an OD_600_ of 2.0 at 37 °C in LB, and 0.2 mL cultures were directly added to 6 mL NFDM medium containing 20 mM ammonium (nitrogen-repression conditions) or without ammonium (nitrogen-fixation conditions) in 10 mL serum bottles, respectively. These cultures were sealed and incubated at 30 °C for 20 h with constant shaking (200 rpm). The cell densities of EN-01 under anaerobic nitrogen-fixation or nitrogen-repression conditions after 20 h reached 3.43 × 10^8^ CFU/mL and 5.42 × 10^8^ CFU/mL, respectively, indicating similar growth status. The EN-01 cells were then centrifuged at 12,000 g for 5 min. Subsequently, cell pellets were rapidly frozen in liquid nitrogen and stored at −80 °C. Total RNA was isolated using TRIzol Reagent (Invitrogen, Carlsbad, CA). To avoid possible DNA contamination, an additional DNase I digestion was performed at 37 °C for 30 min, and the samples were chilled on ice. Total RNA was purified using RNeasy columns, resuspended in RNase-free water and quantitated on a NanoVue plus.

### Microarray analysis

For microarrays, standard methods were used for cDNA synthesis, fragmentation, and terminal biotin labeling based on Affymetrix protocols. Labeled cDNA was hybridized to the Affymetrix *E*. *coli* Genome 2.0 array. Hybridized arrays were stained with streptavidin-phycoerythrin using an Affymetrix Fluidic Station. After staining, arrays were scanned with an Affymetrix GeneChip Scanner 3000 based on the total signal intensity. The resulting microarray data were analyzed using Affymetrix software (MAS 5.0). Consensus “detection p-values”, “change p-values”, and “mean expression ratios” were calculated. All signal intensities with mean expression ratios above 2 were considered significant changes if the p-value was below 0.05. Complete microarray data have been deposited in the Gene Expression Omnibus (GEO) database under accession number GSE37780.

### Quantitative real-time PCR

To investigate the expression of NFI genes in *E*. *coli* EN-01, real-time quantitative PCR (qPCR) was performed using an ABI 7500 Real-Time PCR System. Primers pairs used for qPCR were designed using DNAman and are listed in Table S6. RNA was extracted from cells treated under the same conditions described for the microarray analyses. Reverse transcription was performed using a cDNA synthesis kit (Promega, USA). SYBR Green premix was used to detect PCR amplification. The 16S RNA gene was used to normalize the results. Additionally, qPCR was also used to verify microarray results.

### Proteome analysis by two-dimensional PAGE

As previously described, *E*. *coli* EN-01 cells were cultured under the anaerobic nitrogen-fixation or nitrogen-repression conditions. The cells were harvested via high-speed centrifugation (12,000 g, 5 min and 4 °C). Cell pellets were washed three times with PBS buffer and suspended in lysis buffer (8 M urea, 2 M thiourea, 0.5% w/v CHAPS, 2% v/v carrier ampholyte, 1% w/v DTT and 1 mM PMSF) for sonication on ice. The samples were centrifuged at 14000 g, 4 °C for 30 min, then, the supernatant was taken for the Bradford Protein assay and frozen at −80 °C.

The details of two-dimensional protein gel electrophoresis and 2-DE analysis were performed according to the protocol described by Zhengfu Zhou *et al*.^61^. Briefly, an equal amount of total protein extract (1000 µg) was isoelectrically separated on a 17 cm immobilized pH gradient strip (Bio-Rad, USA) with a linear pH gradient from 4 to 7. Separation along the second-dimension was performed using vertical 12% gels, followed by Coomassie Brilliant Blue G-250 staining visualization and image acquisition. Gels were scanned with a PowerLook 1000 (UMAX Technologies Inc., Dallas, TX) and the gel images were analyzed with PDQuest V7.3.0 (Bio-Rad Laboratories) software according to the manufacture’s protocol. The protein spots with intensity levels greater than 2.0 or less than 0.5 were picked and subjected to mass spectrometric (MS) analysis.

For MALDI-TOF MS analysis, the protein spots with significant difference were excised from the gels for tryptic digestion as described^61^. The peptides were analyzed on a 4700 Proteomics Analyzer (Applied Biosystems, USA). Proteins were identified using GPS Explorer software V3.5 (Applied Biosystems) and the function was quoted from the National Center for Biotechnology Information (http://www.ncbi.nlm.nih.gov) database.

### Measurement of intercellular organic acid content

*E*. *coli* DH10B and its recombinant strain EN-01 were cultured under anaerobic nitrogen-fixation conditions, and cells were collected at 0 h and 45 h. A1501 was grown under microaerobic nitrogen-fixation conditions, and the bacteria were centrifuged at 0 h and 10 h. The cell suspension was centrifuged at 12000 g, 4 °C for 10 min, and the supernatant was used to analyze the organic acid content. Organic acid concentrations were determined using an HPLC (1200 series, Agilent Technologies) equipped with a Bio-Rad HPX-87H column with 5 mM H_2_SO_4_ as the mobile phase (0.6 ml/min, 50 °C).

## Electronic supplementary material

Supplementary materials
