# Supplementary material for: Global investigation of an engineered nitrogen-fixing Escherichia coli strain reveals regulatory coupling between host and heterologous nitrogen-fixation genes
Source: Sci Rep. 2018 Jul 19;8:10928. doi: 10.1038/s41598-018-29204-0 (PMC6053447; doi:10.1038/s41598-018-29204-0)
Supplement: Supplementary file 1 — Supplementary materials [file 41598_2018_29204_MOESM1_ESM.pdf]

Supplementary Information

Global investigation of an engineered nitrogen-fixing *Escherichia coli* strain reveals regulatory coupling between host and heterologous nitrogen-fixation genes

Zhimin Yang[1,2], Yunlei Han[2], Yao Ma[2], Qinghua Chen[2], Yuhua Zhan[2], Wei Lu[2], Li Cai[1], Mingsheng Hou[1], Sanfeng Chen[3], Yongliang Yan[2,*] & Min Lin[2,*]

Supplementary Figures

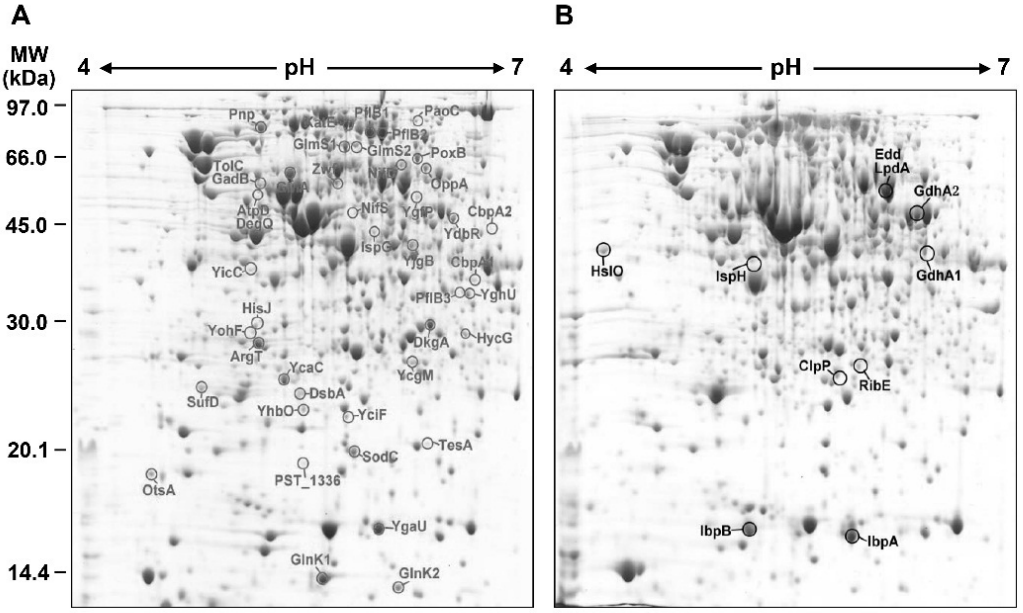

Figure S1. Differential protein synthesis patterns of *E. coli* EN-01 under nitrogen-fixation conditions.

Coomassie Blue stained the two-dimensional protein gels of total protein from nitrogen-fixation conditions and nitrogen excess conditions in parallel. Circles indicate protein spots differentially expressed between the two conditions. (A) Total protein under nitrogen-fixation condition. Gray circles, proteins induced under this condition. (B) Total protein under nitrogen-excess conditions. Black circles, proteins repressed under nitrogen-fixation conditions. The circled spot changes were observed in three repeat experiments. Proteins were identified by MALDITOF-MS analysis.

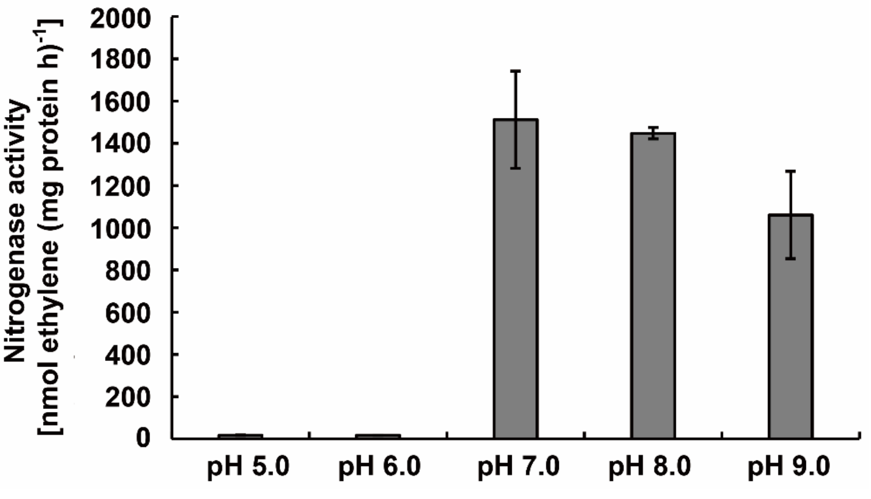

Figure S2. *P. stutzeri* A1501 nitrogenase activity under microaerobic conditions when cells were cultured in minimal medium without nitrogen with varying pH.

All experiments were performed three times.

Supplementary Tables

Table S1. Genes with similar expression patterns in *E. coli* EN-01 and *P. stutzeri* A1501 under nitrogen-fixation and nitrogen-excess conditions.

Gene	EN-01		A1501		Product
	Fold change	ID	Fold change	ID	
**Upregulated**					
glnK	86.88	b0450	6.85	PST0502	nitrogen regulatory protein P-II
glgA	11.68	b3429	2.23	PST2137	glycogen synthase
iciA	3.66	b2916	2.03	PST3130	chromosome initiation inhibitor
osmC	5.81	b1482	3.86	PST0265	osmotically inducible protein OsmC
katE	15.47	b1732	2.27	PST0039	catalase
amtB	55.28	b0451	2.55	PST0504	ammonium transporter
amtB	55.28	b0451	3.86	PST0503	ammonium transporter
yeaG	20.34	b1783	2.07	PST0722	serine protein kinase PrkA
glnL/ntrB	10.36	b3869	2.86	PST0350	nitrogen regulation protein NtrB
glnG/ntrC	10.61	b3868	3.19	PST0349	nitrogen regulation protein NtrC
ynfM	26.45	b1596	2.4	PST0565	putative transport protein YnfM
rutA	22.56	b1012	10.74	PST3597	hypothetical protein
rutB/ycdL	16.73	b1011	7.91	PST3598	isochorismatase family protein ycdL
**Downregulated**					
fpr	-2.41	b3924	-2.04	PST1518	ferredoxin--NADP reductase
acnB	-2.19	b0118	-2.36	PST2046	aconitate hydratase 2
fdx	-2	b2525	-2.07	PST3037	ferredoxin
accC	-2.48	b3256	-2.52	PST0186	acetyl-CoA carboxylase, biotin carboxylase
accB	-2.05	b3255	-2.68	PST3273	biotin carboxyl carrier protein (BCCP)
infA	-2.91	b0884	-5.05	PST2297	translation initiation factor IF-1
queA	-2.9	b0405	-2.14	PST3052	S-adenosylmethionine:trna ribosyltransferase-isomerase
topA	-2.24	b1274	-2.19	PST1729	DNA topoisomerase I
creA	-8.28	b4397	-2.41	PST0960	CreA family protein
secG	-2.95	b3175	-3.05	PST3315	preprotein translocase, SecG subunit
phoP	-4.81	b1130	-2.02	PST0389	two-component response regulator PhoP

Table S2. Up-regulated and down-regulated proteins in *E. coli* EN-01 under nitrogen-fixation conditions compared with nitrogen-excess conditions.

Protein function [a]	Protein name [a]	ORF no. [a]	Fold change [b]	Transcriptional ratios [c]	Experimental pI/MW(kDa)	EN01-1 [d]	EN01-2 [d]	EN01- 3 [d]	EN01-NH_4^+- 1[e]	EN01-NH_4^+- 2 [e]	EN01-NH_4^+- 3 [e]
**Upregulated**											
**Energy production and conversion**											
xanthine dehydrogenase family protein, molybdopterin-binding subunit	PaoC	b0284	4.51	2.64	6.28 /89.43	10677.52	11983.71	8080.42	2473.21	2272.84	2064.76
formate acetyltransferase	PflB	b0903	9.07	3.61	5.89 /82.08	79820.07	407578.31	309101.28	33823.87	17139.11	36831.71
F0F1 ATP synthase subunit beta	AtpD	b3732	Present [f]	4.35	5.01 /57.70	7653.83	5997.1	5206.97	0	0	0
hydrogenase 3 and formate hydrogenase complex, HycG subunit	HycG	b2719	Present [f]	24.80	6.64 /29.10	19537.53	20459.2	13388.17	0	0	0
**Amino acid transport and metabolism**											
glutamine synthetase	GlnA	b3870	2.03	7.61	5.26 /61.97	289832.63	37147.68	118378.93	57434.42	80955.52	80633.23
cystine transporter subunit	FliY	b1920	2.31	-1.64	5.06 /27.32	20701.87	35823.57	57987.79	17916.95	18431.98	13205.36
oligopeptide ABC transporter, periplasmic oligopeptide-binding protein	OppA	b1243	2.36	2.47	6.36 /66.12	40116.44	46987.46	33417.05	16323.41	20914.87	13926.92
pyruvate dehydrogenase	PoxB	b0871	2.43	4.87	6.30 /70.51	65159.8	79142.16	52377.86	24985.06	27228.57	28608.11

histidine ABC transporter, periplasmic histidine-binding protein	HisJ	b2309	4.15	9.19	4.98 /29.54	36150.55	26584.92	22719.2	6428.93	9904.91	4274.55
asparagine synthetase B	AsnB	b0674	4.79	1.46	5.55 /67.49	49417.91	78407.34	62968.32	14520.67	15260.93	15743.42
multifunctional acyl-CoA thioesterase I and protease I and lysophospholipase L1	TesA	b0494	Present [f]	3.44	6.38 /20.45	6190.78	9500.38	7482.43	0	0	0
peptidase B	PepB	b2523	Present [f]	0.90	5.61 /49.92	12920.84	26648.82	11392.6	0	0	0
glutamate decarboxylase isozyme	GadB	b1493	Present [f]	25.14	5.01 /59.64	31924.04	27675.11	17496.52	0	0	0
putative transport protein	YdcS	b1440	Present [f]	1.82	6.64 /43.66	29133.55	27857.91	36215.28	0	0	0
predicted oxidoreductase, Zn-dependent and NAD(P)-binding	YbdR	b0608	Present [f]	3.08	6.57 /48.83	30937.75	45376.91	27138.29	0	0	0
nitrogen regulatory protein P-II 2	GlnK	b0450	Present [f]	86.88	6.14 /14.03	58043.93	68251.67	42932.55	0	0	0
selenocysteine synthase	SelA	b3591	2.16	1.12	6.44 /58.27	8310.03	9456.8	6966.4	5634.95	1923.29	3904.54
lysine-arginine-ornithine-binding periplasmic protein	ArgT	b2310	Present [f]	35.73	5.00 /27.90	212837.19	119965.22	105171.81	0	0	0
**Nucleotide transport and metabolism**											
deoxyribose-phosphate aldolase	DeoC	b4381	3.76	1.19	5.70 /29.02	76300.9	79586.59	74272.4	25008.19	17381.48	18838.22
guanine deaminase	YgfP	b2883	Present [f]	2.07	6.30 /55.73	14275.27	8858.43	15291.98	0	0	0
**Carbohydrate transport and metabolism**											
alpha,alpha-trehalose-phosphate synthase	OtsA	b1896	2.19	14.34	4.20 /18.84	26775.55	35598.46	28366.25	20257.74	11660.89	9577.1

phosphopyruvate hydratase	Eno	b2779	2.78	1.79	5.46 /50.13	238485.81	335433.31	243209.45	138489.92	108317.13	47516.21
glyceraldehyde-3-phosphate dehydrogenase	GapA	b1779	2.71	-1.01	6.81 /38.17	301419.25	252047.86	269862.31	64522.14	43432.99	195470.06
glycerol-3-phosphate transporter periplasmic binding protein	UgpB	b3453	Present [f]	0.85	6.40 /49.58	13698.9	19931.49	16862.25	0	0	0
glucose-6-phosphate 1-dehydrogenase	Zwf	b1852	Present [f]	2.00	5.63 /61.05	16686.32	8395.39	54276.13	0	0	0
**Coenzyme transport and metabolism**											
nicotinamide-nucleotide adenylyltransferase	NadR	b4390	Present [f]	1.78	5.61 /49.92	12920.84	26648.82	11392.6	0	0	0
glycogen branching enzyme	GlgB	b3432	2.09	3.52	6.41 /86.12	7467.03	7851.56	5053.94	2240.98	2915.56	4582.13
**Lipid transport and metabolism**											
4-hydroxy-3-methylbut-2-en-1-yl diphosphate synthase	IspG	b2515	Present [f]	2.19	5.93 /45.28	8618.21	10410.06	8816.28	0	0	0
predicted oxidoreductase with NAD(P)-binding Rossmann-fold domain	YohF	b2137	Present [f]	3.11	4.94 /28.94	24960.61	9695.64	9657.03	0	0	0
**Translation, ribosomal structure and biogenesis**											
polynucleotide phosphorylase/polyadenylase	Pnp	b3164	3.80	3.03	5.03 /84.20	36250.27	92173.78	95961.63	11099.99	41358.55	6600.91
selenocysteine-specific translation elongation factor	SelB	b3590	2.14	1.51	6.60 /77.51	6848.07	9243.59	5522.94	3440.22	3434.1	3220.38
**Transcription**											

DNA-binding transcriptional regulator CsiR	CsiR	b2664	Present [f]	4.20	6.73 /25.68	4297.85	4668.76	3109.85	0	0	0
**Cell wall/membrane/envelope biogenesis**											
L-glutamine:D-fructose-6-phosphate aminotransferase	GlmS	b3729	2.38	3.79	5.70 /76.99	83182.41	105947.39	81678.21	28565.22	38858.22	46462.52
outer-membrane lipoprotein carrier protein	LolA	b0891	3.12	-2.01	5.71 /21.04	23890.07	25584.98	22370.58	11224.33	7815.73	4003.94
outer membrane protein TolC	TolC	b3035	Present [f]	2.51	5.01 /59.64	31924.04	27675.11	17496.52	0	0	0
**Posttranslational modification, protein turnover, chaperones**											
cysteine desulfurase activator complex subunit	SufD	b1681	Present [f]	2.92	6.90 /55.84	6544.38	5925.19	4065.63	0	0	0
glutathione S-transferase YghU	YghU	b2989	3.22	5.68	6.68 /33.76	38692.62	34271.93	26826.43	9706.74	11543.7	9724.58
curved DNA-binding protein CbpA	CbpA	b1000	Present [f]	2.80	6.96 /35.52	32523.53	23415.64	23012.08	0	0	0
periplasmic protein disulfide isomerase I	DsbA	b3860	4.32	3.24	5.39 /24.01	55759.93	29482.02	52869.19	10653.49	10870.27	10440.11
serine peptidase DegQ	DegQ	b3234	Present [f]	2.47	5.01 /57.70	7653.83	5997.1	5206.97	0	0	0
conserved protein	YbeL	b0643	Present [f]	-1.10	5.04 /18.04	22022.03	12809.27	9323.94	0	0	0
**Inorganic ion transport and metabolism**											
DNA protection during starvation protein	Dps	b0812	3.87	1.53	6.08 /19.52	79388.73	75383.64	74407.08	21475.36	15061.94	22691.99
hydroperoxidase II	KatE	b1732	2.94	15.47	5.70 /88.15	95539.57	72345.97	57848.5	12035.92	21784.89	42976.7
superoxide dismutase	SodC	b1646	Present [f]	2.94	5.76 /19.95	114789.43	108092.42	88218.53	0	0	0
thiosulfate transporter subunit	CysP	b2425	Present [f]	1.15	6.82 /35.63	20128.07	13530.59	24539.7	0	0	0

**Secondary metabolites biosynthesis, transport and catabolism**											
predicted hydrolase	YcaC	b0897	3.00	3.36	5.22 /25.00	144939.47	97448.28	141008.89	49178.2	34413.05	44121.52
fumarylacetoacetate hydrolase family protein	YcgM	b1180	Present [f]	2.63	6.26 /26.24	21708.94	19513.85	20872.69	0	0	0
**Signal transduction mechanisms**											
putative sensor-like histidine kinase YfhK	YfhK	b2556	Present [f]	1.65	6.17 /67.51	18207.94	16189.23	9317.45	0	0	0
two-component response regulator	ArcA	b4401	2.09	-5.26	5.23 /29.69	39667.48	62332.28	82985.18	31040.23	34255.95	23194.07
DNA-binding transcriptional regulator CpxR	CpxR	b3912	2.69	-1.06	5.52 /29.19	22428.25	18636.93	14576.23	5213.51	8048.4	7435.03
**General function prediction only**											
quercetinase activity in vitro	YhhW	b3439	2.29	-0.94	5.18 /32.82	21831.43	10486.6	19326.6	11612.08	6623.29	4357.37
2,5-didehydrogluconate reductase A	DkgA	b3012	3.44	2.07	6.40 /29.46	92496.3	106684.42	86604.19	25515.1	35565.52	21959.24
periplasmic protein	OsmY	b4376	20.31	-1.14	5.36 /21.41	151591.67	118169.62	171191.2	6721.91	10278.41	4706.62
predicted oxidoreductase with NAD(P)-binding Rossmann-fold domain	YhhX	b3440	Present [f]	1.65	6.52 /48.13	15423.54	10153.25	5881.94	0	0	0
oxidoreductase, zinc-binding dehydrogenase family	YjgB	b4269	Present [f]	2.09	6.24 /42.12	47531.85	39563.63	41745.54	0	0	0
predicted intracellular protease	YhbO	b3153	Present [f]	7.50	5.38 /22.84	64092.88	29909.31	67314.84	0	0	0
**Function unknown**											
hypothetical protein	YigA	b3810	2.21	-1.01	5.47 /22.72	25648.86	15764.65	16649.71	8065.59	9922.33	8329.08

conserved protein	YicC	b3644	2.11	2.88	4.97 /38.15	22058.33	10631.31	9523.56	5006.41	6441.15	8573.92
predicted protein	YgaU	b2665	5.06	5.62	5.96 /16.34	245207.84	253176.19	234093.84	52365.71	45143.2	47247.07
putative structural proteins	YciF	b1258	Present [f]	2.62	5.71 /22.35	19012.46	20388.78	18002.55	0	0	0
**Nitrogen fixation island**											
nitrogenase metalloclusters biosynthesis protein NifS	NifS [g]	PST1351	Present [f]	51.3	5.78 /53.42	6963.21	14656.29	3414.32	0	0	0
MoFe protein, alpha subunit	NifD [g]	PST1327	Present [f]	32.4	6.17 /67.51	18207.94	16189.23	9317.45	0	0	0
Predicted dehydrogenases and related proteins	PST1336 [g]	PST1336	Present [f]	39.6	5.38 /19.38	24088.09	8742.44	19414.71	0	0	0
**Downregulated**											
**RNA processing and modification**											
oligoribonuclease	Orn	b4162	-3.03	-1.18	4.73 /24.43	9098.19	14068.15	9330.53	36131.91	29164.15	33109.08
**Energy production and conversion**											
dihydrolipoamide dehydrogenase	LpdA	b0116	-2.99	-2.75	6.18 /57.58	56598.43	127645.84	25557.87	254452.25	57837.09	315874.13
phosphoenolpyruvate carboxykinase (ATP)	Pck	b3403	absent	-1.17	5.59 /68.04	0	0	0	36430.63	38259.81	48119.72
predicted oxidoreductase	YbdH	b0599	-3.58	1.42	5.90 /43.78	8774.57	10979.1	8114.74	30006.88	43497.24	26280.76
oxygen-insensitive NAD(P)H nitroreductase	NfnB	b0578	-2.16	1.54	5.96 /26.37	28804.92	41919.6	26287.59	53080.47	143290.31	12978.92
**Amino acid transport and metabolism**											
acetolactate synthase I, large subunit	IlvB	b3671	absent	0.73	5.30 /68.75	0	0	0	38016.14	73289.27	35485.25

threonine deaminase	IlvA	b3772	absent	-1.55	5.69 /63.73	0	0	0	24481.38	28379.67	38401.97
O-succinylhomoserine (thiol)-lyase	MetB	b3939	absent	1.14	6.45 /44.68	0	0	0	24239.77	34008.5	24223.99
glutamate dehydrogenase, NADP-specific	GdhA	b1761	-7.40	-2.83	6.44 /52.06	29211.08	30002.09	30655.72	277676.16	217072.55	170477.48
5,10-methylenetetrahydrofolate reductase	MetF	b3941	-8.69	1.96	6.26 /30.84	7299.61	7103.46	4704.97	11787.5	93968.05	60224.76
dihydroxyacid dehydratase	IlvD	b3771	-8.00	1.02	5.81 /74.30	13936.62	15733.89	6622.83	101661.64	96741.55	92073.8
branched-chain amino acid aminotransferase	IlvE	b3770	-5.22	-1.53	5.56 /35.82	18327.35	21701.1	14432.3	63254.97	117118.41	103894.3
shikimate kinase I	AroK	b3390	-2.41	1.86	5.37 /20.38	16142.32	8690.47	18526.2	33093.54	41515.46	30049.91
cysteine synthase A	CysK	b2414	-2.35	-1.71	5.93 /36.78	14016.04	18586.75	13035.83	34556.94	37422.72	35246.63
phospho-2-dehydro-3-deoxyheptonate aldolase	AroG	b0754	-2.74	-1.44	6.61 /41.26	36909.09	31800.61	35035.91	94650.8	117022.41	72191.32
**Nucleotide transport and metabolism**											
deoxycytidine triphosphate deaminase	Dcd	b2065	-2.40	-1.33	5.89 /26.07	9384.1	10606.33	8500.86	23506.97	21230.95	23630.9
nucleoside diphosphate kinase	Ndk	b2518	-4.11	-1.27	5.82 /16.17	22277.23	26338.69	22684.02	102015.91	105794.41	85167.12
adenine phosphoribosyltransferase	Apt	b0469	-2.30	1.89	5.29 /24.80	30153.17	29483.78	38498.63	75689.65	75418.02	74900.48
**Carbohydrate transport and metabolism**											
6-phosphogluconate dehydrogenase	Gnd	b2029	-2.44	1.05	5.03 /52.58	88572.82	47881.77	70907.34	233766.09	69702.33	203984.17

galactose-1-epimerase (mutarotase)	GalM	b0756	-4.06	1.19	4.52 /46.18	4161.61	7899.85	4001.38	26761.48	19006.74	19442.58
dihydroxyacetone kinase subunit M	DhaM	b1198	-2.37	1.74	4.28 /61.44	2577.76	5994.25	3071.64	12839.21	7310.6	7472.04
**Coenzyme transport and metabolism**											
riboflavin synthase subunit alpha	RibE	b0415	-2.54	-5.56	5.94 /25.86	12778.35	14581.08	11278.82	38269.34	31827.47	28207.88
GTP cyclohydrolase II	RibA	b1277	-2.56	-1.86	5.90 /24.91	11777.34	11443.32	8796.5	29363.88	27978.73	24642.35
S-adenosylmethionine synthetase	MetK	b2942	-2.82	1.13	4.93 /50.27	26681.05	29343.18	20474.42	90838.11	33628.05	91264.6
glutamate-1-semialdehyde aminotransferase	HemL	b0154	-3.38	1.24	4.41 /49.33	6183.41	8573.31	8262.15	36710.5	23063.72	18065.61
pyridoxamine kinase	PdxY	b1636	absent	1.49	6.53 /30.40	0	0	0	8598.31	9861.19	8770.25
**Lipid transport and metabolism**											
4-hydroxy-3-methylbut-2-enyl diphosphate reductase	IspH	b0029	-2.33	-2.10	5.13 /39.24	3669.75	4141.6	3417.17	10769.77	7088.45	8333.4
**Translation, ribosomal structure and biogenesis**											
tyrosyl-tRNA synthetase	TyrS	b1637	-2.22	1.24	5.84 /50.21	37375.21	58452.7	31579.82	81569.73	82705.83	118206.37
ribosome recycling factor	Frr	b0172	-3.08	1.27	5.91 /24.41	7840.33	8898.43	8490.84	31657.99	26231.36	19713.46
50S ribosomal protein L7/L12	RplL	b3986	-3.18	1.39	4.28 /14.56	47803.42	66588.53	63704.26	229539.64	171419.98	165383.13
methionyl tRNA synthetase	MetG	b2114	-3.61	2.44	5.79 /80.87	10972.85	33312.61	4419	52287.39	59628.54	64036.46
leucyl-tRNA synthetase	LeuS	b0642	-2.28	2.71	5.11 /96.46	7332.34	9844.02	10359.16	21477.2	20512.73	20778.93
**Transcription**											
transcriptional repressor MprA	MprA	b2684	-5.29	-1.05	6.15 /21.06	8752.04	1545.35	6165.73	29917.84	29967.06	27190.46

transcriptional regulator kdgR	IclR	b4018	-6.48	1.20	5.44 /32.09	24074.2	16642	27680.72	150553.41	151445.09	141386.81
transcription antitermination protein NusG	NusG	b3982	absent	1.80	6.38 /21.60	0	0	0	6977.88	8770.56	5386.64
DNA-binding transcriptional regulator OxyR	OxyR	b3961	-2.22	1.84	6.47 /37.29	19712.31	12220.15	11013.55	28567.06	36083.06	30754.15
**Cell wall/membrane/envelope biogenesis**											
alanine racemase	Alr2	b4053	absent	-1.08	6.85 /44.53	0	0	0	4928.02	5321.1	4894.04
3-deoxy-manno-octulosonate cytidylyltransferase	KdsB	b0918	-2.00	1.17	5.09 /32.68	23208.32	10632.43	12552.83	42314.24	28138.5	22163.18
glucose-1-phosphate thymidylyltransferase	RfbA	b2039	-3.04	1.53	5.53 /31.73	16907.38	23722.25	17684.05	73826.11	51039.49	52174.43
GDP-mannose 4,6-dehydratase	Gmd	b2053	absent	1.62	6.14 /46.98	0	0	0	25036.07	5950.76	8019.78
outer membrane protein A	OmpA	b0957	-2.50	1.86	5.90 /18.97	11913.64	9947.09	7138.24	26625.68	23994.02	21946.15
D-alanyl-alanine synthetase A	DdlB	b0092	-3.02	3.52	4.80 /49.47	11585.98	11187.74	8210.42	38246.95	33031.5	22161.79
**Posttranslational modification, protein turnover, chaperones**											
ATP-dependent Clp protease proteolytic subunit	ClpP	b0437	absent	-2.38	5.77 /25.10	0	0	0	20241.49	10250.34	15811.18
heat shock protein IbpA	IbpA	b3687	-15.46	-4.33	5.88 /16.01	14930.61	16020.35	8425.25	288347.63	49751.41	270715.31
alkyl hydroperoxide reductase subunit C	AhpC	b0605	-5.36	-1.44	4.82 /23.40	63761.68	87968.7	142460.13	272085.38	834374.38	471427.28
Hsp33-like chaperonin	HslO	b3401	-3.18	-16.38	4.02 /41.23	13268.13	19124.97	13699.24	36743.54	49281.96	60575.85
alkyl hydroperoxide reductase, F52a subunit, FAD/NAD(P)-binding	AhpF	b0606	-2.08	-0.95	5.60 /60.81	24979.74	51586.84	28670.05	73367.01	53821.85	91792.66

peroxiredoxin	Bcp	b2480	-2.02	3.09	4.87 /18.25	29997.48	24790.01	14579.71	61259.92	55028.6	23709.66
heat shock chaperone IbpB	IbpB	b3686	-2.02	-5.71	5.11 /15.88	63345.59	157825.98	155647.95	282802.44	259774.81	217381.23
**General function prediction only**											
ClpXP protease specificity-enhancing factor	SspB	b3228	-3.75	-1.56	4.16 /26.45	7673.35	10968.57	12966.14	24000.53	53391.7	41024.33
modulator of drug activity B	MdaB	b3028	-3.17	-1.83	6.26 /23.94	5124.2	4836.35	4320.23	17199.98	14016.38	14083.69
global DNA-binding transcriptional dual regulator H-NS	Hns	b1237	absent	-1.34	5.57 /17.03	0	0	0	23813.91	28365.97	24136.01

a Protein names, accession numbers and descriptions are from EcoCyc database. The data are grouped according to their biological function.

b Translation level ratios (cells under nitrogen-fixation conditions/cells under nitrogen-excess conditions).

c Transcriptional ratios obtained from DNA microarray experiments (see Materials and Methods) are indicated.

d Date set of EN01-1, EN01-2 and EN01-3 was the gray intensity of protein from three repetitions under nitrogen-fixation conditions.

e Date set of EN01-NH_4^+-1, EN01-NH_4^+-2 and EN01-NH_4^+-3 was the the gray intensity of protein from three repetitions under nitrogen-excess conditions.

f Absent: all proteins were detectable only under nitrogen-fixation conditions and were absent under nitrogen-excess conditions.

g Protein-coding genes located on the nitrogen fixation island.

Table S3. Transcriptionally altered genes in recombinant *E. coli* strain EN-01 under nitrogen-fixation conditions compared with the nitrogen-excess conditions.

Gene name	Locus_tag	Change ratio	Functional description
**nitrogen regulatory system genes and nitrogen assimilation related genes**			
rpoN	b3202	3.64	RNA polymerase, sigma 54 (sigma N) factor
ntrB	b3868	10.61	nitrogen regulation protein NR(I), *glnG*
ntrC	b3869	10.36	nitrogen regulation protein NR(II), *glnL*
glnB	b2553	2.12	regulatory protein P-II for glutamine synthetase
glnK	b0450	86.88	nitrogen assimilation regulatory protein for GlnL, GlnE, and AmtB
amtB	b0451	55.28	ammonium transporter
glnA	c4819	7.61	glutamine synthetase
glnD	b0167	5.27	PII uridylyl-transferase / uridylyltransferase
glnH	b0811	2.74	glutamine ABC transporter periplasmic protein
glnP	b0810	9.04	glutamine transporter subunit
glnQ	b0809	9.16	glutamine ABC transporter ATP-binding protein
gltB	b3212	14.16	glutamate synthase, large subunit
gltD	b3213	7.49	glutamate synthase, 4Fe-4S protein, small subunit
gdhA	b1761	-2.83	glutamate dehydrogenase
nac	b1988	124.98	nitrogen assimilation transcriptional regulator
gltI	b0655	3.56	glutamate and aspartate transporter subunit
gltJ	b0654	3.84	glutamate and aspartate transporter subunit
gltK	b0653	5.27	glutamate and aspartate transporter subunit
gltL	b0652	4.18	glutamate and aspartate transporter subunit
gltP	z5676	11.78	glutamate/aspartate:proton symporter
gadC	b1492	12.19	predicted glutamate/gamma-aminobutyric acid antiporter
hisM	b2307	10.45	histidine/lysine/arginine/ornithine transporter subunit M
hisP	b2306	4.52	histidine/lysine/arginine/ornithine transporter subunit P
hisQ	b2308	10.30	histidine/lysine/arginine/ornithine transporter subunit Q
lysP	b2156	3.31	lysine transporter
argT	b2310	35.73	lysine/arginine/ornithine transporter subunit
hisJ	b2309	9.19	histidine transport system histidine-binding periplasmic protein
ygjU	b3089	2.34	sodium:serine/threonine symporter
potC	b1124	2.03	polyamine transporter subunit
potD	b1123	2.18	polyamine transporter subunit
gsiB	b0830	4.77	predicted peptide transporter subunit: periplasmic-binding component of ABC superfamily
oppA	b1243	2.47	oligopeptide transporter subunit
oppC	b1245	2.17	oligopeptide transporter subunit
oppD	b1246	3.09	oligopeptide transporter subunit
gsiC	b0831	2.02	predicted peptide transporter subunit: membrane component of ABC superfamily

ddpA	b1487	7.49	putative hemin-binding lipoproteinputative hemin-binding lipoprotein
dppA	b3544	6.89	dipeptide transporter
dppB	b3543	2.07	dipeptide/heme transporter
dppD	b3541	2.26	dipeptide/heme transporter
gsiA	b0829	2.34	glutathione transporter ATP-binding protein, ABC superfamily
sapA	b1294	2.41	antimicrobial peptide transport ABC transporter periplasmic binding protein
sapB	b1293	2.54	antimicrobial peptide transport ABC transporter permease
sapC	b1292	2.45	antimicrobial peptide transport ABC transporter permease
sapD	b1291	2.8	antimicrobial peptide transport ABC system ATP-binding protein
astD	b1746	17.79	succinylglutamic semialdehyde dehydrogenase
astE	b1744	14.33	succinylglutamate desuccinylase
astB	b1745	16.68	succinylarginine dihydrolase
astC	b1748	41.67	succinylornithine transaminase, PLP-dependent
astA	c2147	36.61	arginine succinyltransferase
gabD	b2661	2.3	succinate-semialdehyde dehydrogenase I, NADP-dependent
gabP	b2663	2.53	gamma-aminobutyrate transporter
gabT	b2662	5.61	4-aminobutyrate aminotransferase, PLP-dependent
fklB	ECs5185	3.46	peptidyl-prolyl cis-trans isomerase
codA	c0456	1.16	cytosine deaminase
codB	b0336	1.63	cytosine transporter
nupC	b2393	1.43	nucleoside (except guanosine) transporter
ydcS	b1440	1.82	predicted spermidine/putrescine transporter subunit
ydcT	b1441	1.80	putative ATP-binding component of a transport system
ydcU	b1442	1.83	predicted spermidine/putrescine transporter subunit
ydcV	c1867	1.58	predicted spermidine/putrescine transporter subunit
ydcW	b1444	-1.11	gamma-aminobutyraldehyde dehydrogenase
ycdG	b1006	13.1	pyrimidine permease
ycdF	b1005	4.94	hypothetical protein
**Central carbon pathways related genes**			
pgi	b4025	3.40	glucose-6-phosphate isomerase
fbp	b4232	1.63	fructose-1,6-bisphosphatase
tpiA	b3919	-1.15	triosephosphate isomerase
fba	b2097	2.33	fructose-bisphosphate aldolase
gapC	b1417	2.20	glyceraldehyde 3-phosphate dehydrogenase
pgk	b2926	1.14	phosphoglycerate kinase
gpmA	b0755	-1.21	phosphoglycerate kinase

eno	b2779	1.79	phosphopyruvate hydratase
pykF	b1676	8.28	pyruvate kinase
ldhA	b1380	-4.18	D-lactate dehydrogenase
adhE	b1241	3.12	bifunctional acetaldehyde-CoA/alcohol dehydrogenase
pta	b2297	4.27	phosphate acetyltransferase
ackA	b2296	2.54	acetate kinase
aceE	b0114	-3.01	pyruvate dehydrogenase subunit E1
aceF	z0125	-2.11	dihydrolipoamide acetyltransferase
lpdA	b0116	-2.75	lipoamide dehydrogenase
pflB	z1248	5.44	formate acetyltransferase 1
fdhF	b4079	6.59	selenopolypeptide subunit of formate dehydrogenase H
ppc	b3956	10.64	phosphoenolpyruvate carboxylase
maeB	b2463	-5.36	predicted phosphotransacetylase
gltA	z0873	-2.73	type II citrate synthase
acnB	z0128	-3.29	bifunctional aconitate hydratase 2, 2-methylisocitrate dehydratase
icdA	b1136	1.31	isocitrate dehydrogenase
frdA	b4154	-3.56	fumarate reductase flavoprotein subunit
aceA	b4015	-2.15	isocitrate lyase
aceB	b4014	-5.69	malate synthase
mdh	b3236	-1.16	malate dehydrogenase
zwf	b1852	2.02	glucose-6-phosphate 1-dehydrogenase
gnd	c2556	1.15	6-phosphogluconate dehydrogenase
rpiA	b2914	1.27	ribose-5-phosphate isomerase A
rpiB	b4090	1.23	ribose-5-phosphate isomerase B
tktA	b2935	2.72	transketolase
tktB	b2465	17.14	transketolase
talA	b2464	7.64	transaldolase
edd	b1851	-2.34	phosphogluconate dehydratase
eda	b1850	-2.85	keto-hydroxyglutarate-aldolase
appA	b0980	2.74	phosphoanhydride phosphorylase
appB	b0979	3.37	cytochrome bd-II oxidase subunit II
appC	b0978	7.63	cytochrome bd-II oxidase subunit I
hyaA	b0972	7.98	hydrogenase-1 small subunit
hyaB	b0973	5.71	hydrogenase 1 large subunit
hyaC	b0974	5.32	hydrogenase 1 b-type cytochrome subunit
hyaD	b0975	4.81	hydrogenase 1 maturation protease
hyaE	b0976	2.52	hydrogenase-1 operon protein HyaE
hyaF	b0977	2.27	hydrogenase-1 operon protein HyaF
fhlA	b2731	2.15	DNA-binding transcriptional activator
hycA	b2725	13.41	formate hydrogenlyase regulatory protein HycA
hycB	b2724	19.03	formate hydrogenlyase subunit 2
hycC	b2723	13.51	formate hydrogenlyase subunit 3

hycD	b2722	15.02	membrane-spanning protein of hydrogenase 3
hycE	b2721	14.89	formate hydrogenlyase subunit 5 precursor
hycF	b2720	18.84	formate hydrogenlyase complex iron-sulfur subunit
hycG	b2719	24.8	formate hydrogenlyase complex iron-sulfur subunit
hycH	b2718	9.82	formate hydrogenlyase maturation protein
ndh	b1109	2.41	respiratory NADH dehydrogenase 2/cupric reductase
napH	b2204	2.04	ferredoxin-type protein essential for electron transfer from ubiquinol to periplasmic nitrate reductase (NapAB)
narG	b1224	2.26	nitrate reductase 1, alpha subunit
narH	b1225	2.29	nitrate reductase 1, beta (Fe-S) subunit
narI	b1227	2.02	nitrate reductase 1, gamma (cytochrome b(NR)) subunit
narJ	b1226	3.05	molybdenum-cofactor-assembly chaperone subunit (delta subunit) of nitrate reductase 1
narW	b1466	2.33	nitrate reductase 2 (NRZ), delta subunit (assembly subunit)
narY	b1467	2.63	nitrate reductase 2 (NRZ), beta subunit
narZ	b1468	3.77	nitrate reductase 2 (NRZ), alpha subunit
narU	b1469	3.63	nitrate/nitrite transporter
nirD	b3366	3.03	nitrite reductase, NAD(P)H-binding, small subunit
norR	b2709	4.92	Anaerobic nitric oxide reductase DNA-binding transcriptional activator
tatB	b3838	2.44	TatABCE protein translocation system subunit
tatA	b3836	2.49	TatABCE protein translocation system subunit
tatE	b0627	4.43	TatABCE protein translocation system subunit
pflB	b0903	5.44	formate acetyltransferase 1
adhP	c1911	3.56	alcohol dehydrogenase
yjgB	b4269	2.09	predicted alcohol dehydrogenase
**Oxygen protection related genes**			
sodC	b1646	2.94	superoxide dismutase, Cu, Zn
katE	b1732	15.47	hydroperoxidase HPII(III) (catalase)
ccmD	b2198	4.13	cytochrome c biogenesis protein
nrfA	b4070	2.24	nitrite reductase, formate-dependent, cytochrome
osmC	b1482	5.81	Lipoyl-dependent Cys-based peroxidase, hydroperoxide resistance; salt-shock inducible membrane protein; peroxiredoxin
yhbO	b3153	7.5	stress-resistance protein
dps	b0812	1.53	DNA starvation/stationary phase protection protein Dps

Table S4. Peak area of organic acids in the medium from *E. coli* EN-01 and *P. stutzeri* A1501 under nitrogen-fixation conditions.

organic acids	*E. coli* DH10B		*E. coli* EN-01		*P. stutzeri* A1501	
	0 h	45 h	0 h	45 h	0 h	10 h
Pyruvic acid	0	1222.4	0	560.9	0	252.4
Succinic acid	0	231.1	0	212.2	0	0
Lactic acid	0	675.8	0	366.1	3228.6	3047
Formic acid	0	372.8	0	404.7	0	0
Acetic acid	0	389.9	0	422.1	0	0

Table S5. Bacterial strains and plasmids used in this study.

Bacterial Strains	Genotype or description	Reference or source
E.coli		
DH10B	Wild-type strain of *E. coli*	Laboratory stock
EN-01	*E. coli* DH10B strain bearing the NFI of *Pseudomonas stutzeri* A1501	Han *et al.*
Pseudomonas stutzeri		
A1501	Wild-type strain of *Pseudomonas stutzeri*	Laboratory stock

Table S6. List of primers used in this study.

Gene name	Primers	Sequence (5' - 3')
PST1302	PST1302F	CGCATACGCCAGCAACTCG
	PST1302R	CCGCTGTTCTTCAATGCCTC
PST1303	PST1303F	TCAAGAGCACGCCAAATCA
	PST1303R	TGACCGTGCTCGCAACAGA
nifQ	PST1304F	GAACAGGACGGCGGCTAC
	PST1304R	TGCTCTTCTCCAAAACAATC
PST1305	PST1305F	TCAACGGGCCTGGATGG
	PST1305R	CGACTACGCCGAGCAACA
nifB	PST1306F	CCTGGATCTACTGGAACAAC
	PST1306R	ATGCGGCTGACCTCTTT
rnfB	PST1316F	ATTCTGGTTCTTGCACTGAT
	PST1316R	CGCCTCGATTTCCTTGAT
rnfC	PST1317F	CCCTCGCACATTCCCTT

	PST1317R	TGCTTGACGTAATCGTTCTT
rnfD	PST1318F	CGCTGGTCTTCGTCATT
	PST1318R	ACCTGGCGGGTCCACTTCG
rnfG	PST1319F	TCCAGTTCATCGCCCTCG
	PST1319R	CCACCGTCCTTCTTCACC
rnfE	PST1320F	CGGCAGCGGCACGTTGTT
	PST1320R	CCAGCACGAAGCCCAGA
rnfH	PST1321F	CTGAGCAAGCAAAAGGTC
	PST1321R	CGTCGTCATCGTCGTCT
nifY2	PST1322F	CATCGCCTCCAACAACG
	PST1322R	GGAGACCACATAGAGCACC
PST1325	PST1325F	ACCAGAGCGAGGTCAATCCC
	PST1325R	ATGCCGAGGAACAGGTAGCG
nifH	PST1326F	GATGATGGCGATGTATGC
	PST1326R	TCGGTGTTGCGGCTGTT
nifT	PST1329F	CAAGAAGGACCAGGAAGAA
	PST1329R	AGGGTGATCGGCAGTTT
nifY	PST1330F	CTCTACACCCTGAGCATCG
	PST1330R	GGCCGCATGACCTTGAC
PST1331	PST1331F	AGCGAGCACCCTGGTATCC
	PST1331R	GCAGTGCTGGCTGGAGTTGA
PST1332	PST1332F	CTGTTCCGCTTCATCCGC
	PST1332R	GCTTTCCTCAGGCAGACGCT
nifE	PST1333F	TACCAAGAACCTCGGCAACC
	PST1333R	CGATGTTGTATTCACCGATCAG
nifN	PST1334F	GCGTTCGCCAAGGTGTTCT
	PST1334R	GCTGCTTGTCGCAAATGGT
nifX	PST1335F	ACCAGCATTTCGGTTCGTC
	PST1335R	TTGTCCTCGTTGCCGTCCT
PST1336	PST1336F	GGCACCTACGACACCTGG
	PST1336R	TCGCCGACGATGGGGAT
PST1341	PST1341F	GCGATTTCCTCGGCAGCAT
	PST1341R	CGAACTCGGCATAGACCTG
PST1342	PST1342F	CAGCATCGCCAACAAGACC

	PST1342R	CACCCTCGGCAATCACCC
PST1344	PST1344F	TTCCAGTCATCGCAACAGAG
	PST1344R	CGGCGGATAGAGCAGGT
modC	PST1345F	CTGGAAGTCAACGGCGAACG
	PST1345R	TCACCACATGGTCCCAGTCC
modB	PST1346F	GTGGGCTCGGTGCTTTACT
	PST1346R	ACGCTGAAGAAGGTGTCCC
modA	PST1347F	AGCCGCTTCACCTATGCCA
	PST1347R	GCGGTCTAAGGTTTCCAGTG
PST1348	PST1348F	ATGCAGGCCGAATACGAGC
	PST1348R	ATTGATTGCGTGCGCTGGT
nifU	PST1350F	GTGACGTCGGCTCGCTGAG
	PST1350R	CTCGTCGACGGTCAGCCC
nifS	PST1351F	TGAACAAGGTCGGCATCG
	PST1351R	TGTCCATCGCACGCATAA
nifV	PST1352F	CGATACGGTGGGAGTAATGG
	PST1352R	CGTTGACCGTGGTGTTGATAT
cysE	PST1353F	GCACGGTGGTCGGTATCC
	PST1353R	GGTCGGGAATCAGGTAATGG
PST1354	PST1354F	TGTTCGGCGAGGGCAAGG
	PST1354R	GGGGAAAGGTGAAAATGGC
nifW	PST1355F	GCGCTGGAAGAGCTGGTAT
	PST1355R	AGGGCACGCCGAAGAAGTT
nifZ	PST1356F	GTCTGCCGCTCTACGAATATG
	PST1356R	CAGCCCTGATCGAGGAAAT
nifM	PST1357F	CTGGGCTTTCACCTGCTGT
	PST1357R	TGGTGCCGCTCGTATTGAC
PST1358	PST1358F	CCTACATGCAGCATCTGGACG
	PST1358R	CTTCTCCAGCTCGACCGTTC
nifF	PST1359F	GTTTCTCGATGCGATGGG
	PST1359R	TGAGGTTGCTCTGGTTGTCC
16s	16SF	CCTACGGGAGGCAGCAG
	16SR	ATTACCGCGGCTGCTGG
